# *In situ* structures of the contractile nanomachine myophage Mu in both its extended and contracted states

**DOI:** 10.1128/jvi.02056-24

**Published:** 2025-02-24

**Authors:** Junquan Zhou, Liwen Wang, Hao Xiao, Wenyuan Chen, Zhonghua Liu, Jingdong Song, Jing Zheng, Hongrong Liu

**Affiliations:** 1 Institute of Interdisciplinary Studies, Key Laboratory for Matter Microstructure and Function of Hunan Province, Key Laboratory of Low-Dimensional Quantum Structures and Quantum Control, School of Physics and Electronics, Hunan Normal University 12568 , Changsha, China; 2 The National & Local Joint Engineering Laboratory of Animal Peptide Drug Development, College of Life Sciences, Hunan Normal University 554899 , Changsha, China; 3 National Institute of Pathogen Biology, Chinese Academy of Medical Sciences and Peking Union Medical College 220736 , Beijing, China; Michigan State University, East Lansing, Michigan, USA

**Keywords:** phage Mu, extended and contracted states, DNA circularization protein, contractile injection systems, cryo-EM

## Abstract

Myophage Mu is a representative of contractile nanomachines with a simple tail baseplate. It has the capacity to infect a range of intestinal bacteria and has extensive applications in genetic engineering research. Nevertheless, a comprehensive understanding of the entire structure and contractile mechanisms of Mu remains elusive. Using cryo-electron microscopy (cryo-EM), we resolved the asymmetric structures of Mu in both its extended and contracted states, the latter of which lacked the tail baseplate, at near-atomic resolutions. We built the atomic models for the extended Mu, encompassing the head, the connector complex, the tail, and the simple baseplate. It is noteworthy that we identified the position and structure of the tail tube initiator protein gp43 (referred to as the DNA circularization protein). The protein gp43 plays a crucial role not only in the baseplate assembly and DNA circularization but also in stabilizing the wedge-hub connection and mediating tail contraction. Except for the baseplate structure, the structural comparison of Mu in its extended and contracted states revealed that only the tail sheath protein gp39 and the C-terminus of the tail terminator protein gp37 undergo notable conformational changes to accommodate the tail contraction, whereas the remaining protein components remained unchanged. Our structures exhibited conserved properties among the majority of myophages, thereby providing valuable insights into the contraction mechanisms across myophages and contractile injection systems (CISs).

**IMPORTANCE:**

Despite extensive study, the asymmetric structures of phage Mu, a highly effective transposable myophage, remain unknown. In this study, we present the high-resolution structures of Mu in both its extended and contracted states. The comparison of the two structures allows for the illustration of detailed conformational changes of the head-to-tail complex during the process of tail contraction. The contraction mechanism of Mu is highly conserved and widely adapted to all contractile nanomachines that share common structural features with Mu.

## INTRODUCTION

Myophages are distinguished by a complex, long, and contractile tail that is more elaborate than that of siphophages and podophages. Additionally, Myophages possess larger genomes on average (1, 2). The tail components of myophages typically comprise a tail tube, a tail sheath, a tape measure protein (TMP), and a baseplate (3). These components exhibit structural similarities with contractile injection systems (CISs), which are phage tail-like nanomachines. Examples of such systems include R-type pyocins (4, 5), bacterial type VI secretion system (T6SS) (6, 7), and *Photorhabdus* virulence cassettes (PVC) (8). Upon binding to the surface of the host cell, the baseplate undergoes a significant conformational change, resulting in the contraction of the tail sheath. This contraction drives the rigid tube to penetrate the cell envelope and inject DNA into the cytoplasm (9). The structure and function of the *Escherichia coli* T4 phage have been extensively studied as a model system for myophages (9–12). In contrast to T4, which possesses a highly complex baseplate (12), several recently reported myophages, including phage Pam3 (13), E217 (14), XM1 (15), and Milano (16), have a much simpler tail baseplate. The simple baseplates of these myophages exhibit a striking structural similarity, playing a pivotal role in tail assembly, host attachment, membrane penetration, and contraction-coupled genome ejection (13–15). However, notable discrepancies remain in the structural specifics of the intricate protein components that comprise these simple baseplates (14, 16, 17). Furthermore, only a limited number of myophages with a simple baseplate in their contracted state have been resolved to near-atomic resolution (14), and the precise conformational changes of related protein components remain poorly understood upon contraction. Consequently, the contraction mechanism of myophages with a simple baseplate remains to be further elucidated.

Myophage Mu is a temperate phage (18). Upon infecting a host cell, the majority of temperate phages insert their genome into one or a limited number of specific sites within the host chromosome. In contrast, the genome of phage Mu can be randomly integrated into the host’s chromosome, resulting in host mutations and the induction of various genome rearrangements (19, 20). Phage Mu is a highly effective transposable phage that employs both replicative and non-replicative transposition mechanisms (21, 22). It is an ideal model for the study of DNA transposition and has significant potential for the development of genetic engineering tools and host evolution (23, 24). Phage Mu is distinguished by two sets of interchangeable fiber and fiber assembly genes, which represent a unique characteristic. In particular, the expression of these genes is regulated by the orientation of the G segment, including G(+) type expressing gp49 and gp50, and G(−) type expressing gp51 and gp52, which enables Mu to infect not only *E. coli* but also other intestinal bacteria (25–27). Moreover, phage Mu also contains a specific functional N protein (gp43), which accompanies the DNA into the host bacteria and converts linear DNA into circular DNA (28–31). However, the precise location and structure of gp43 remain unknown. Previous studies for the Mu on the assembly pathway and composition (32), as well as X-ray analysis (27, 33–36), proposed that phage Mu has a simple baseplate, which may share similar structural properties with those of phage Pam3 (13) and E217 (14). However, the function and properties of phage Mu remain poorly understood due to the lack of a complete atomic structure.

Using cryo-EM, we resolved the three-dimensional (3D) structures of the G(+) type phage Mu in both its extended state and urea-induced contracted state, the latter lacking the baseplate, at near-atomic resolutions. The high-resolution density maps enabled us to identify almost all proteins from the head to the tail, including the major capsid protein (MCP) gp34, the connector complex (gp29, gp36, and gp37), the tail (gp39, gp40, and gp42), and the baseplate (gp43, gp44, gp45, gp46, gp47, gp48, and gp49). Comparison of the structures of Mu in the extended and contracted states reveals that the connector complex and the tail tube remain unchanged, with the exception of the C-terminus of the tail terminator gp37, which extends to accommodate the contraction of the sheath. The sheath undergoes significant conformational changes, resulting in a notable compaction and contraction of the sheath and the exposure of the rigid tail tube. The structures of Mu provide insights into the conformational dynamics of the contractile nanomachine with a simple baseplate and offer insights into the infection and contraction mechanisms of myophages and CISs.

## RESULTS

### Overall structures of myophage Mu in extended and contracted states

**Fig 1:**
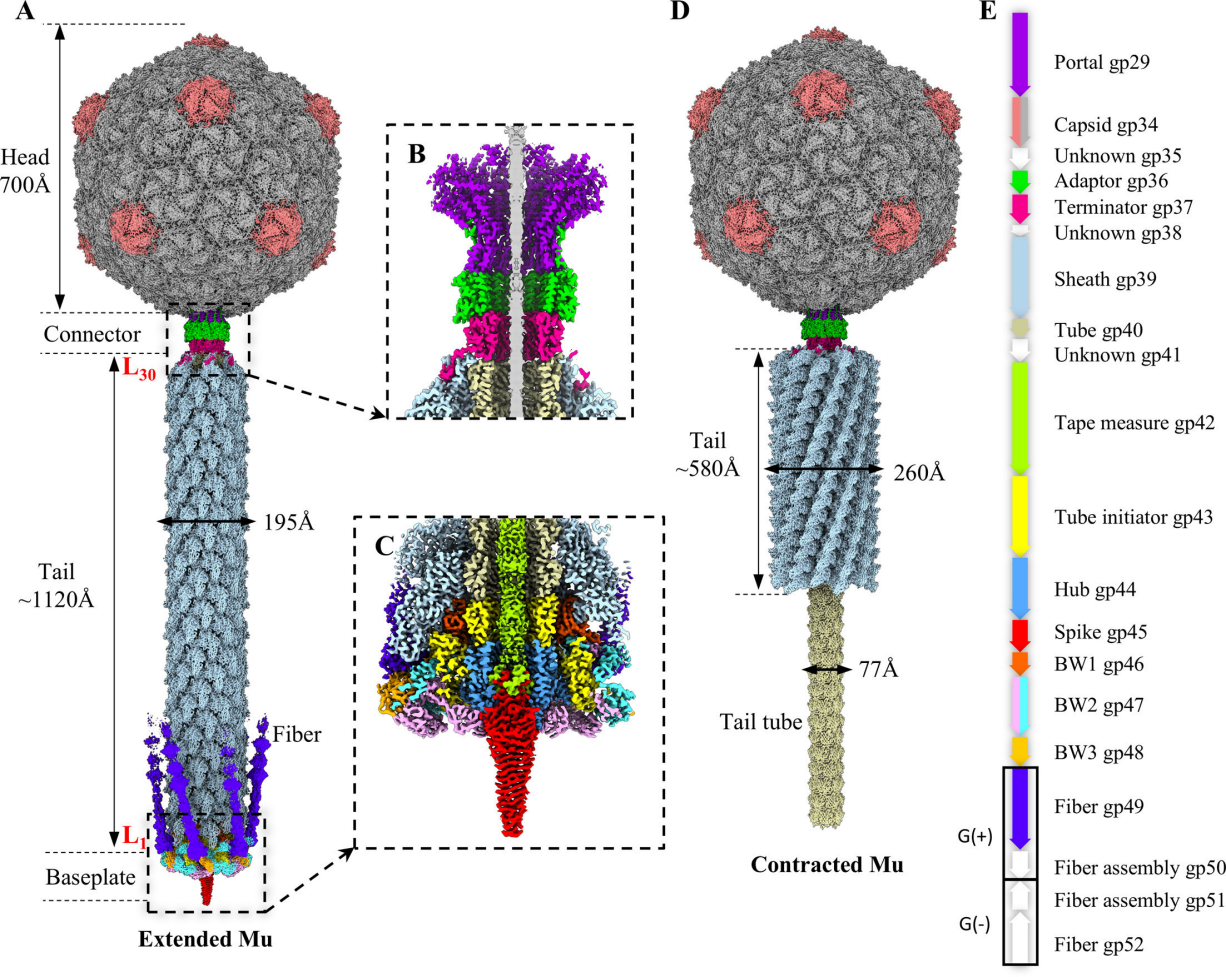
Overall structures of phage Mu in both extended and contracted states. (**A**) Side view of the asymmetric structure of extended Mu. L_1_ and L_30_ denote the initial (first) and final (30th) layers of the tail sheath, respectively. (**B, C**) Cut-open view of the density maps of connector complex (**B**) and baseplate (**C**). (**D**) Side view of the asymmetric structure of contracted Mu. (**E**) Scheme of Mu genome segment encoding structural genes. The color code is applied to panels A-E.

Extended Mu was purified from *E. coli* for cryo-EM data collection (Fig. S1A), and contracted Mu was obtained by treating extended Mu samples with urea (Fig. S1B). A total of 3,353 and 5,770 movies were collected for the extended and contracted Mu, respectively. Using icosahedral reconstruction method (37), a 4.2 Å resolution map of the extended Mu head was generated (Fig. S1C). The local reconstruction method (38, 39) was employed to improve the resolution of the 3-fold capsid of the head to 3.2 Å (Fig. S1C). By employing the symmetry mismatch reconstruction (40) and the local reconstruction methods, the structures of the portal-adaptor and the terminator-tail region of extended Mu were resolved to resolutions of 3.4 Å and 3.5 Å, respectively (Fig. S1C). The baseplate was classified and reconstructed by using RELION software (41), generating a final resolution of 3.3 Å (Fig. S1C). The high-resolution density maps allowed us to build the atomic models for the majority of proteins of extended Mu, including the MCP gp34, portal protein gp29, adaptor protein gp36, tail terminator protein gp37, sheath protein gp39, tube protein gp40, the C-terminus of TMP gp42, tube initiator protein gp43, hub protein gp44, spike protein gp45, baseplate wedge protein 1 (BW1) gp46, baseplate wedge protein 2 (BW2) gp47 and baseplate wedge protein 3 (BW3) gp48, and the N-terminus of G(+) type fiber protein gp49 (Fig. 1A; Fig. S2) (42). However, the fiber assembly protein gp50, the C-terminus of fiber gp49, and the N-terminus of TMP gp42 have not been resolved to a high resolution, possibly due to their flexibility. The structural resolution was estimated using the Fourier shell correlation criterion with a cut-off of 0.143 according to the “gold standard” method (Table S1) (43).

The structure of extended Mu can be divided into four distinct regions: the DNA-containing head, the connector complex, the tail, and the baseplate (Fig. 1A). The Mu head is constituted by 415 copies of the MCP gp34, which are organized into 11 pentons and 60 hexons, thereby forming an icosahedral shell with a triangulation number of seven (Fig. 1A). The tail, which is attached to a unique 5-fold vertex of the head by the connector complex, comprises 30 stacked hexameric rings of the tube protein gp40 and the sheath protein gp39 (Fig. 1A and B). The lumen of the tail tube is filled with the TMP gp42 (Fig. 1C; Fig. S2A). The connector complex is comprised of two dodecameric portal protein gp29 and adaptor protein gp36, and one hexameric tail terminator protein gp37 (Fig. 1B). The simple baseplate is comprised of eight protein components, including a hexameric tube initiator (gp43), a trimeric hub (gp44), a trimeric spike (gp45), six wedges (one BW1 gp46, two BW2 gp47, and one BW3 gp48), and six trimeric fibers (gp49 and gp50) (Fig. 1C). Additionally, there are three probably structural proteins (Fig. 1E), including gp35, gp38, and gp41, of which gp35 protein may belong to an additional connector-related protein (33), and gp41 protein may belong to a tail assembly chaperone (19); however, we could not resolve their structures.

The aforementioned methods were employed to reconstruct the structures of the portal-adaptor and the terminator-tail region of the contracted Mu to resolutions of 3.6 Å and 3.8 Å, respectively (Fig. S1C). The high-resolution densities enable us to build the atomic models for the portal protein gp29, adaptor protein gp36, terminator protein gp37, sheath protein gp39, and tube protein gp40 (Fig. S3). The absence of baseplate density in the contracted tail indicates that the baseplate may have detached during the process of tail contraction, potentially due to the effects of excessive urea induction (Fig. 1D). In contrast to the extended Mu, the DNA and the TMP are ejected, and the external tail sheath contracts toward the connector complex, whereas the inner tail tube is exposed in contracted Mu (Figs. S2A and S3A). The gene products and their corresponding protein names are listed in Table S2; Fig. 1E.

### Structure of the capsid

**Fig 2:**
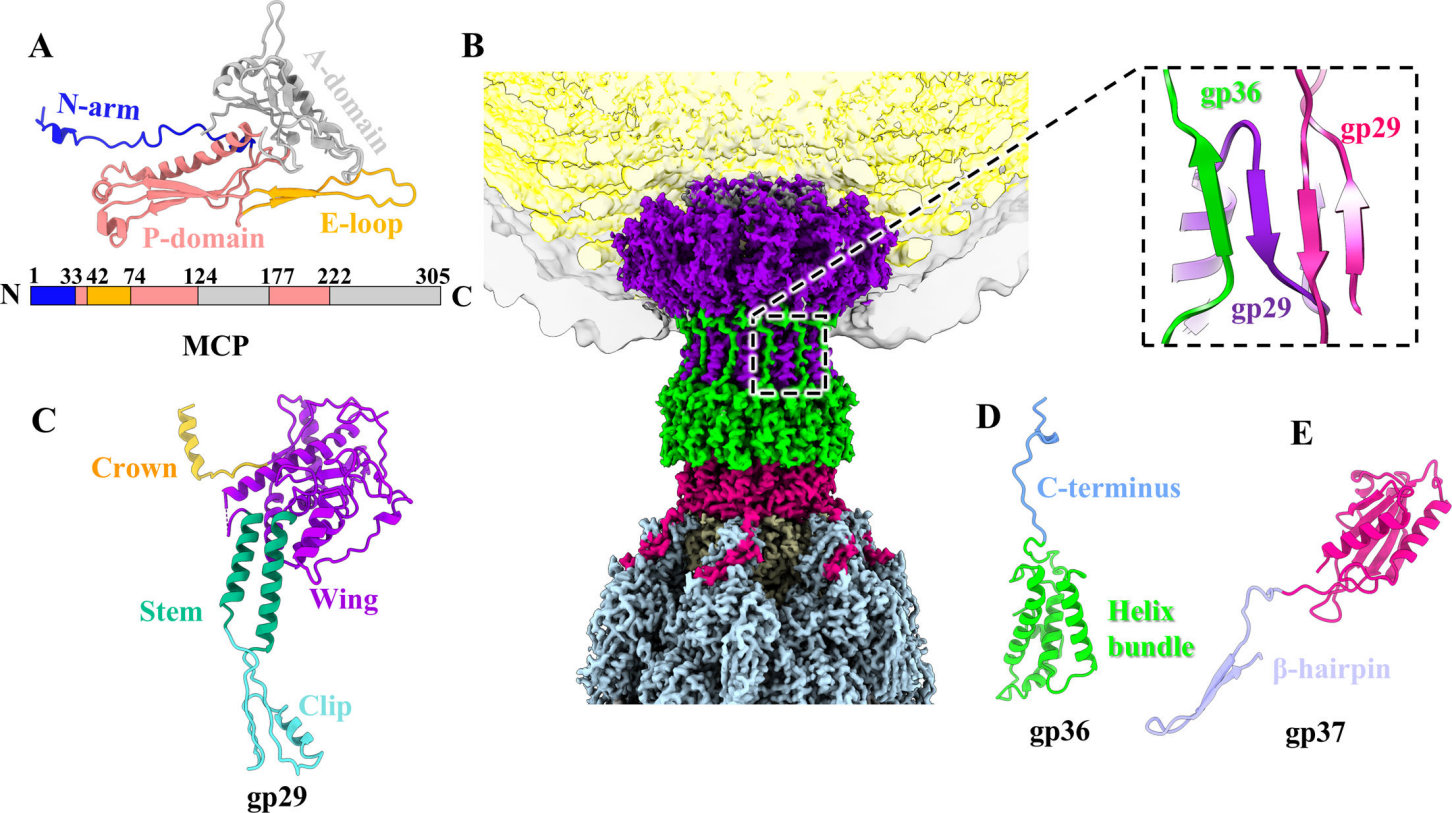
Structures of the head and connector complex of extended Mu. (**A**) Ribbon model of the MCP gp34, shown in four domains. (**B**) Side view of the density maps of the connector complex and cut-open view of the density map of the head. The inset shows a zoomed-in view of the interactions between the C-terminus of the adaptor protein gp36 and the clip domains of two adjacent portal proteins gp29, which form a four-stranded β-sheet. (**C, D**) Ribbon models of the portal protein gp29 (**C**) and the adaptor protein gp36 (**D**), colored according to their domains. (**E**) Ribbon model of the tail terminator protein gp37. The C-terminal β-hairpin domain is colored in lavender.

The MCP gp34 adopts a canonical HK97-like fold (Fig. S4A) (44). According to the domain nomenclature of MCP gp5 of HK97, each gp34 can be divided into four domains (Fig. 2A): N-arm, E-loop, P-domain, and A-domain. An asymmetric unit of the icosahedral head comprises seven gp34 monomers (one hexon and one-fifth of a penton) (Fig. S4B). The superimposition of the seven gp34 monomers in an asymmetric unit revealed considerable conformational changes in the N-arms and E-loops (Fig. S4C), which ultimately led to the formation of capsomers, hexons, and pentons (Fig. S4D) that are assembled into the icosahedral capsid (Fig. 1A). Despite the absence of cement proteins at inter-capsomere interfaces (45), complex and extensive interactions are observed around the 3-fold and the quasi-3-fold axes, including hydrogen bonds, salt bridges, and electrostatic interactions (Fig. S5). Analogous interactions that reinforce the inter-capsomere stability within the head, independent of cement proteins, have been identified in A-1(L) (46), T7 (47), and R4C (48).

### Structure of the connector complex

The Mu connector complex, which attaches the tail to the icosahedral head, is comprised of three proteins: the portal protein gp29, the adaptor protein gp36, and the tail terminator protein gp37 (Fig. 2B). The connector complex in the majority of myophages (13, 14) and siphophages (48, 49) contains the portal, adaptor (the first head-tail joining protein), stopper (the second head-tail joining protein), and tail terminator protein, but Mu is the rarely reported myophage to possess only the first head-tail joining protein.

The Mu portal is constituted of 12 copies of the protein gp29. Each gp29 protein contains four distinct domains: a crown domain, a wing domain, a stem domain, and a clip domain (Fig. 2C). It is noteworthy that the final 124 residues of the C-terminus in gp29 are absent in our structure, which verifies that its C-terminus may undergo proteolytic cleavage by protease I during head assembly (50). The Mu adaptor is constituted by 12 copies of the protein gp36, each of which comprises an α-helix domain and an elongated C-arm (Fig. 2D). The adaptor gp36 exhibits structural similarities to that observed in siphophages (51, 52) and myophages (13, 14, 53) (Fig. S6A and B), with their α-helix domains adopting substantial structural similarity, but their C-arms and β-hairpins exhibiting conformational differences, possibly due to the differences in their structural environment. The C-arm of Mu gp36, which is absent in the X-ray structure (Fig. S6A) (33), is inserted into the clip domain of the portal to form an extended four-stranded β-sheet (Fig. 2B), thereby further stabilizing the interactional interface between the portal and adaptor. The stopper protein usually binds to the tail terminator protein and prevents the release of the genome after it has been packaged into the head (54). Despite lacking the stopper, the adaptor of Mu, which may assume the stopper function, serves as a docking platform by binding to the tail terminator protein to join the head and tail and prevents the release of the genome. A similar phenomenon with only the first head-tail joining protein was also found in siphophages T5 (55) and DT57C (56). The Mu tail terminator protein is constituted by six copies of protein gp37 (Fig. 2E), and the tail sheath and tail tube of Mu are capped by the tail terminator (Fig. 1B), resulting in the termination of the assembly of the tail. Notably, all siphophages (54–56), myophages (13, 14), and CISs (4) encode the tail terminator protein or collar protein, with their main bodies sharing structural similarity, but the C-terminus of myophages and CISs significantly different from that of siphophages (Fig. S6C). Compared with the tail terminator protein of siphophages, which possesses a short C-terminus, the tail terminator protein of myophages and CISs has an extended C-terminus that interacts with tail sheath, forming an enhanced β-sheet (described below). Additionally, all pairwise interfaces between the connector complex and the tube in Mu exhibit complementary electrostatic potential energy (Fig. S7).

### Structure of the tail

**Fig 3:**
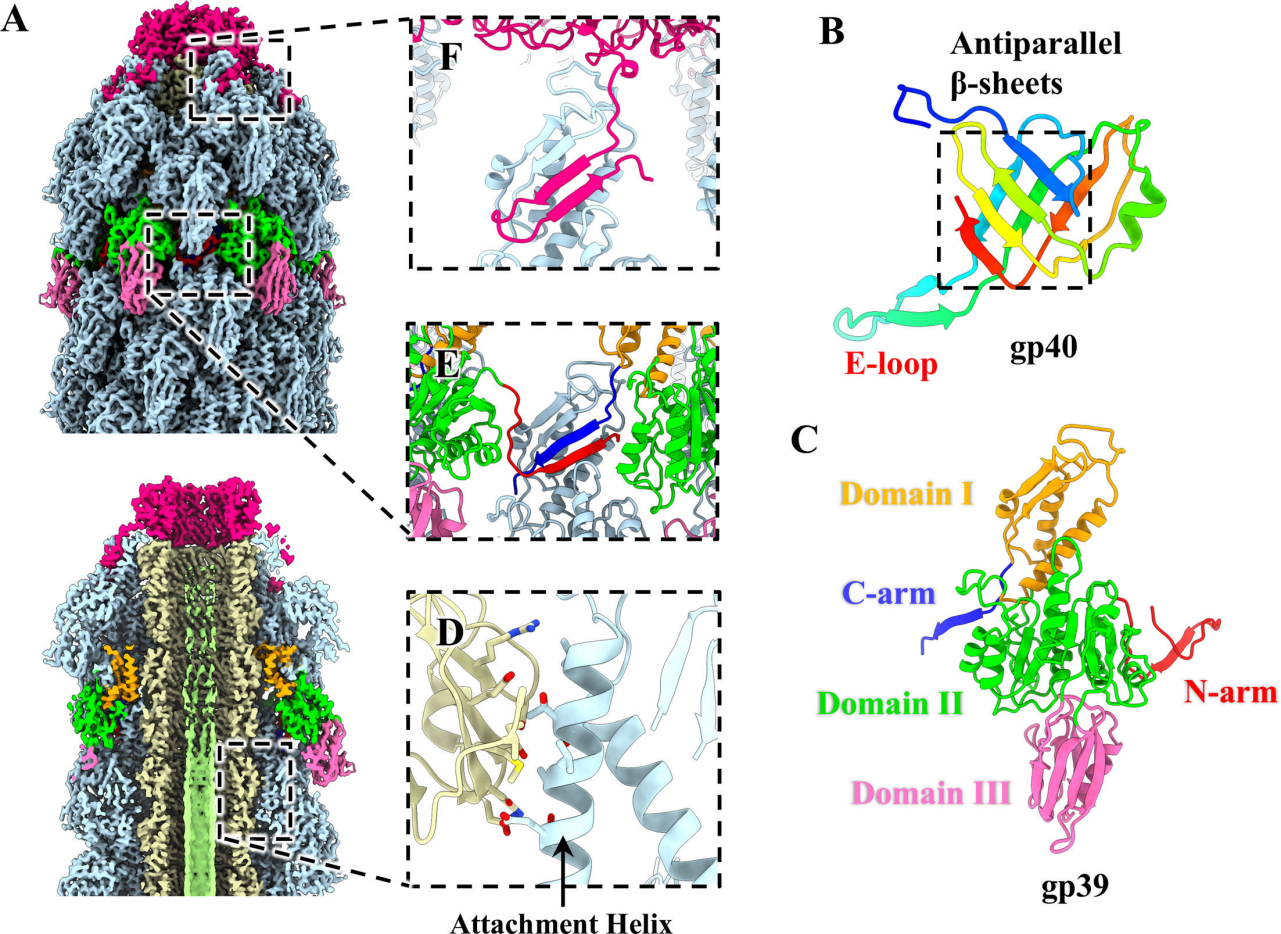
Tail structure of extended Mu. (**A**) Side (upper) and cut-open (bottom) views of the density maps of the tail terminator and tail in extended Mu. The color coding is identical to that used in Fig. 1A, with the exception that the six gp39 monomers of a hexameric ring are colored according to their domains. (**B**) Ribbon model of the tail tube protein gp40, shown in rainbow colors. (**C**) Ribbon model of tail sheath protein gp39, colored according to its domains. (**D**) Zoomed-in view of the interactions between a gp40 monomer (yellow) and an α-helix (residues 72–86) of the domain I in a gp39 monomer. (**E**) Zoomed-in view of the inter-ring and intra-ring interactions among tail sheath monomers. (**F**) Zoomed-in view of the interactions between the domain I of a gp39 monomer in the final layer and the C-terminal β-hairpin of a gp37 monomer (deep pink).

The tail of Mu is comprised of 30 stacked hexameric tail protein rings, with the inner ring consisting of tube protein gp40 and the outer ring comprising sheath protein gp39 (Fig. 1A and 3A). The TMP gp42 is situated within the tail tube (Fig. 3A). The tail tube protein gp40 adopts a conserved β-sandwich structural fold (Fig. 3B), exhibiting topological identity to that observed in myophages (16, 57) and CISs (4, 8). Six copies of gp40 oligomerize to form a continuous 24-stranded β-barrel hexamer (Fig. S8A), with a β-hairpin of each gp40 protruding toward the adjacent ring to mediate the inter-ring interaction (Fig. S8A), which results in the formation of an extended helix tube structure with a helical rise of ~35 Å and a twist of ~25°. In addition, the inner surface of the tube exhibits a remarkably negative charge (Fig. S8B), which facilitates the passage of the DNA along the tube lumen (58). This feature has also been observed in the case of Milano (16) and PVC (8).

The tail sheath protein gp39 can be divided into five domains: N-arm, domain I, domain II, domain III, and C-arm (Fig. 3C). Domain I is responsible for the formation of the sheath inner wall, where an α-helix of the domain I in one tail sheath protein gp39 forms intermolecular contact with one inner tail tube protein gp40 (Fig. 3A and D). In contrast, domain III extends outward (Fig. 3A). The tail sheath proteins of reported myophages (13, 14, 16, 57) and CISs (8, 59, 60) exhibit significant structural divergence in domain III. However, their domains I and II exhibit structural conservation (Fig. S9). Notably, the N-arm of one gp39 monomer and the C-arm of the adjacent monomer from the same sheath ring, together with a two-stranded β-sheet of the domain I from the adjacent sheath ring, form a “handshake” interaction by four-stranded β-sheet across two sheath rings (Fig. 3E), with the exception of the first and last layers of sheath ring. The domain I of the first layer of the sheath ring is linked to the BW1 gp46 (described below), and the domain I of the last layer interacts with the C-terminal β-hairpin domain of gp37 via the formation of an interlaced four-stranded β-sheet (Fig. 3F). The β-sheet augmentations are responsible for the majority of the inter-ring and intra-ring interactions among sheath monomers, which ultimately result in the formation of a right-handed helical structure by all hexameric sheath rings.

### Structure of the central region in the simple baseplate

**Fig 4:**
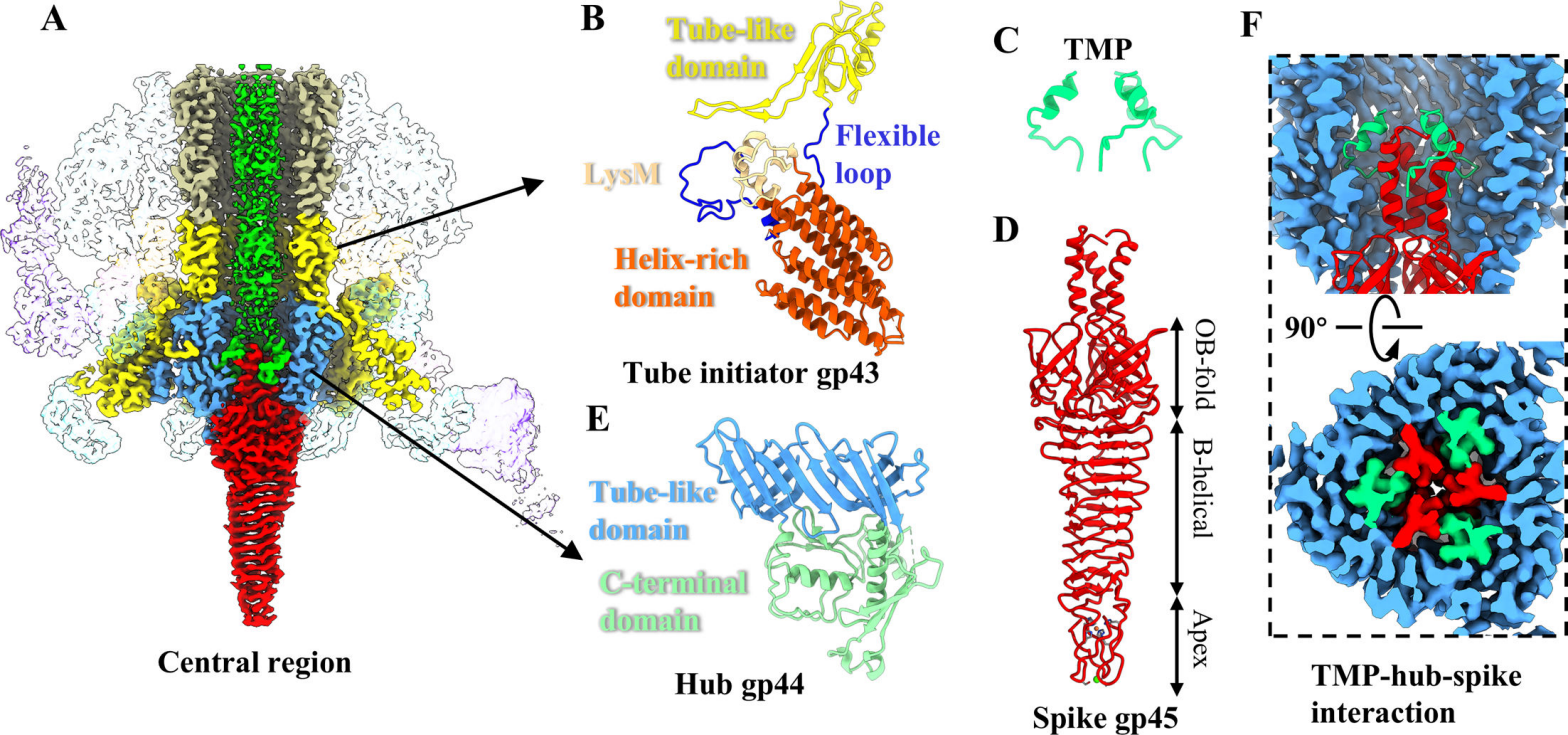
Structures of the central region of the simple baseplate in extended Mu. (**A**) Cut-open view of the density maps of the central region in the simple baseplate. The color coding is identical to that used in Fig. 1A, and the tail sheath and the peripheral region are shown in transparency. (**B**) Ribbon model of the tube initiator protein gp43, colored according to its domains. (**C, D**) Ribbon models of the trimeric C-terminus of TMP gp42 (**C**) and trimeric spike protein gp45 (**D**). (**E**) Ribbon model of the hub protein gp44, colored according to its domains. (**F**) Cut-open (upper) and top (bottom) views of the interactions among the hub, spike, and TMP.

The central region of the simple baseplate of Mu is comprised of the tube initiator (gp43), the hub (gp44), the spike (gp45), and the C-terminus of TMP (gp42) (Fig. 4A). The assembly of the tail tube is initiated by the tube initiator (13), and the hub, spike, and TMP together form a tight interaction to seal the distal tail channel.

The tube initiator protein gp43 is composed of two domains: a tube-like domain and a helix-rich domain, which are connected by a flexible loop (Fig. 4B). The tube-like domains from six copies of gp43 form a 24-stranded β-barrel hexamer, analogous to that of the tube gp40 (Fig. S10A), which serves to initiate the assembly of the tail tube. It is noteworthy that the helix-rich domain comprises eight α-helixes, which extend downward and form extensive interactions with the baseplate wedges (Fig. 4A). Furthermore, a peptidoglycan-binding module (LysM) (61) has been identified in the helix-rich domain of gp43 (Fig. 4B), exhibiting a topologically identical structure to that observed in phages Milano (16) and A-1(L) (17), and CIS R2 pyocin (4) (Fig. S10B). In the majority of reported simple myophages, such as Pam3 (13), XM1 (15), and E217 (14), their tube initiator proteins comprise a conserved β-barrel tube-like structure (Fig. S10B). Additionally, the C-terminal domain of their tube initiator proteins, which extends downward, is connected to a small protein (referred to as the “BH1a” protein [32]) (Fig. S10B). The BH1a protein forms a 6-fold symmetric structure to fill the gaps between the internal hub and external wedge, thus forming a solid baseplate (13–15). It has been reported that the LysM domain and the BH1a protein do not coexist in the majority of myophages with a simple baseplate (32). In the Mu baseplate, the LysM domain of the gp43 helix-rich domain is regarded as a structural component of the tube initiator protein, rather than the BH1a protein. Consequently, it can be deduced that the gp43 helix-rich domain of Mu may be responsible for some of the functions of the BH1a protein. This structural feature has only been previously observed in the baseplate protein gp25 of the phage Milano, which contains the LysM domain but not the BH1a protein (Fig. S10B) (16).

The trimeric TMP gp42 is the determining factor in the length of the tail during the assembly process (19). We only built the atomic model for the C-terminus (residues 674–690) of gp42 (Fig. 4C), and the remainder of gp42 could not be well resolved, likely due to its asymmetric assembly and inherent flexibility within the tail tube. The Mu spike protein gp45 is comprised of three domains: an oligosaccharide-binding (OB-fold) domain, a β-helical domain, and an apex domain (Fig. 4D), which may have the function of binding and penetrating the cell membrane (62). It is noteworthy that two additional spherical densities, which are presumed to be Fe and Ca ions, are present in the apex domain of the spike gp45, which is consistent with the reported X-ray crystal structure (Fig. S11) (35). The presence of these ions is likely to provide the structural stability required for the penetration of the cell envelope. Additionally, Ca ion in Mu is also thought to be associated with membrane binding (35).

The Mu hub is composed of three copies of the protein gp44. Each gp44 contains a tube-like domain and a C-terminal domain (Fig. 4E), which are structurally conserved across the reported myophages (12–14, 16) and CISs (4, 8, 63). The tube-like domains of three gp44 monomers form a pseudo-6-fold symmetric tube-like structure, analogous to that observed in the tube gp40 (Figs. S10A and S12A). Thermodynamic analysis has indicated that the C-terminus of hub gp44 forms a flexible domain (64). Additionally, a comparison of the cryo-EM structure of gp44 with the X-ray crystal structure of gp44 (34) revealed that its C-terminus is slightly constricted inward due to the interaction with the N-terminus of the spike and the C-terminus of the TMP (Fig. S12B). It is hypothesized that conformational changes in the flexible C-terminal domain of gp44 disrupt the interaction sites among TMP, spike, and hub, which may mediate the release of spike gp45 and TMP and lead to gp44 detaching from the baseplate during infection. Furthermore, a previous biochemical experiment demonstrated that the Mu spike protein gp45 exhibited a weak association with the baseplate in the absence of the TMP protein gp42 (32). Our structures provide confirmatory evidence for the tight interaction among the hub, the spike, and the TMP, which is situated at the center of the baseplate (Fig. 4F), suggesting that the stable assembly of the baseplate is inseparable from the TMP. Therefore, our structures, in conjunction with the previous model of the Mu baseplate assembly pathway (32) and structural studies of hub gp44 (34, 64), further complete the assembly of the central region in the baseplate.

### Structure of the peripheral region in the simple baseplate

**Fig 5:**
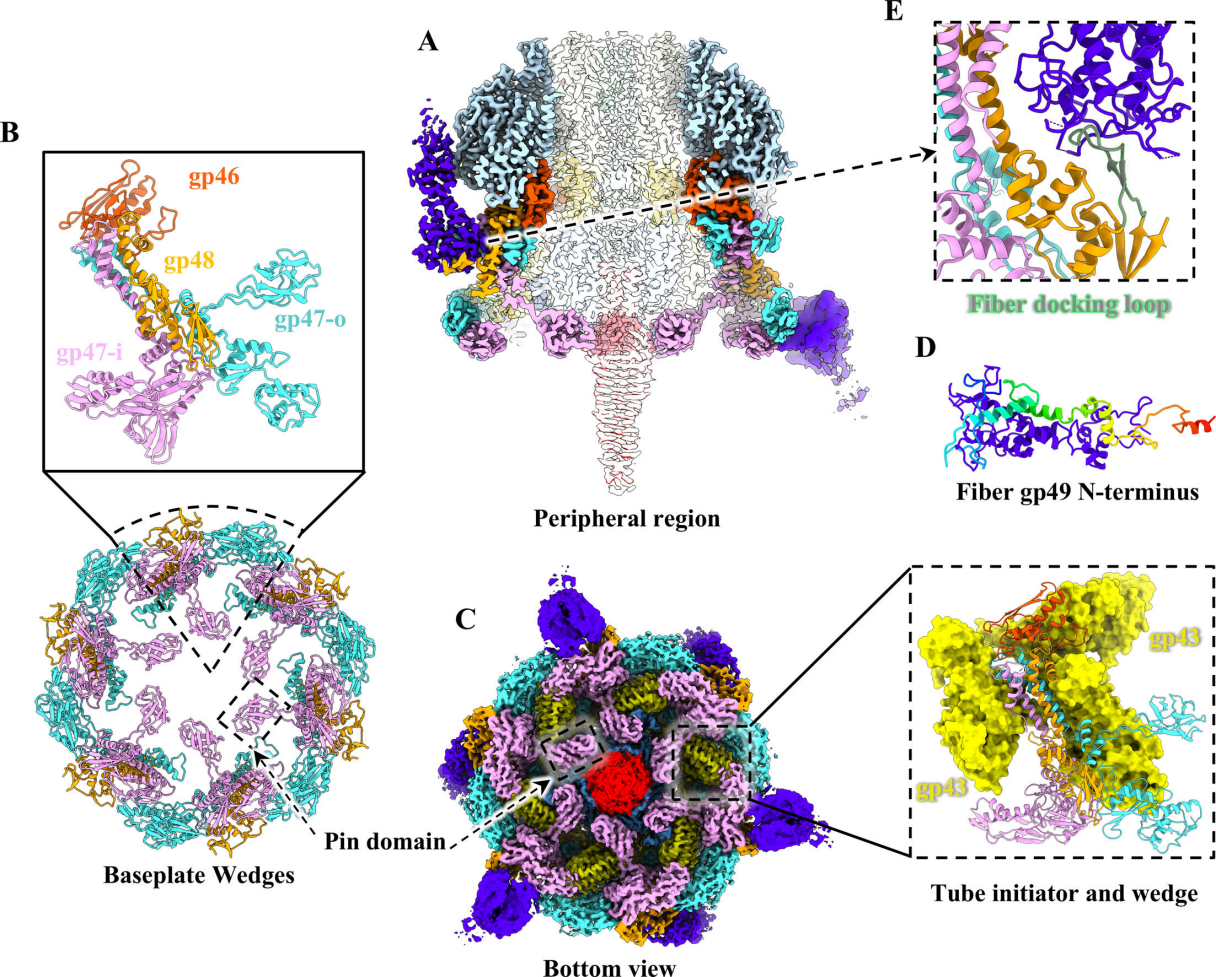
Structures of the peripheral region of the simple baseplate in extended Mu. (**A**) Cut-open view of the density map of the peripheral region in the simple baseplate. The color coding is identical to that used in Fig. 1A, and the central region of the baseplate is shown in transparency. (**B**) Ribbon models of six heterotetrameric wedges and zoomed-in view of the interactions among the baseplate wedge proteins gp46, gp48, gp47-i, and gp47-o. (**C**) Bottom view of the density maps of the simple baseplate and zoomed-in view of the interactions between two tube initiator monomers gp43 and a wedge. (**D**) Ribbon models of the N-terminus of trimeric fiber protein gp49. One of the monomers is colored in rainbow. (**E**) Zoomed-in view of the interactions between the N-terminus of trimeric fiber gp49 and the fiber docking loop of a gp48.

The peripheral region of the simple baseplate is constituted by six baseplate wedges (BW1 gp46, BW2 gp47, and BW3 gp48), and the six G(+) type fibers (fiber gp49 and fiber assembly gp50) (Fig. 5A). The peripheral region is separated from the central region upon contraction of the tail sheath (4, 14).

As with myophages Milano (16), E217 (14), and Pam3 (13), the baseplate wedge of Mu is composed of six heterotetramers, each comprising one BW1 gp46, one BW3, and two conformers of BW2 (referred to as BW2-i and BW2-o) (Fig. 5B). The BW1, comprising six copies of gp46, is situated at the corner between the tube-like domain and the helix-rich domain of the tube initiator protein gp43 (Fig. S13A and B). The monomer of gp46 exhibits structural similarities to the domain I of the sheath protein gp39 (Fig. 5B; Fig. S13A). Notably, the N-arm of one monomer and the C-arm of the adjacent monomer from the first layer of the sheath ring gp39, in conjunction with a two-stranded β-sheet of BW1, form a four-stranded β-sheet to initiate sheath assembly (Fig. S13C). This interaction mediating sheath assembly has been observed in the reported myophages (13–15) and CISs (4, 8, 63).

BW3 is situated at the periphery of the baseplate, whereas BW2-i is anchored to the central region of the baseplate (Fig. 5B). The N-terminal regions of BW3, BW2-i, and BW2-o in a heterotrimer are approximately parallel, collectively forming a core three-helical bundle (Fig. 5B). The apex of this three-helical bundle is in close to the BW1 (Fig. 5B). Moreover, the entire three-helical bundle is embedded between the helix-rich domains of two adjacent tube initiator gp43, thereby stabilizing the BWs into the baseplate (Fig. 5C). The remaining heterotrimer then folds into a trifurcation unit (Fig. 5B). It is noteworthy that BW2-i of a wedge and BW2-o of the adjacent wedge establish a handshaking interaction at the baseplate periphery, which contributes to the primary interaction between two adjacent wedges (Fig. S14A and B). The pin domain of BW2-i is in close proximity to the hub gp44 (Fig. 5C). It is notable that the pin domain of BW2-i monomers exhibits considerable conformational change between adjacent monomers (Fig. S14C and D), which primarily mediate the structural symmetry mismatch between the 6-fold symmetric wedge and the 3-fold symmetric hub.

The six trimeric fibers, which play a crucial role in recognition and binding to host cell lipopolysaccharide (LPS) receptors (65), each comprising fiber protein gp49 and fiber assembly protein gp50, are anchored to the wedge and extend along the groove on the sheath (Fig. S15A and B). Based on our density map, we only built the atomic model for the N-terminus of protein gp49, which is anchored to the fiber docking loop (residues 133–155) of the BW3 (Fig. 5D and E). The density map of the baseplate with 6-fold symmetry imposed indicates that all fibers are docked at the sheath. The crystal structure of the C-terminus of gp49 (36) together with our N-terminus of gp49 can fit perfectly into the fiber map (Fig. S15A and B). The complete structure of fiber can be established, favoring our further exploration of fiber infection. Additionally, the density map of the baseplate with 3-fold symmetry imposed indicates that six tail fibers are attached to the wedge in alternating upward and downward configurations (Fig. S15C), which is consistent with the observations in previous studies of myophages E217 (14) and Pam3 (13). It is hypothesized that the Mu fibers oscillate in an upward and downward motion during infection, thereby facilitating exploration of the receptor LPS.

### Structural changes in the extended and contracted states

**Fig 6:**
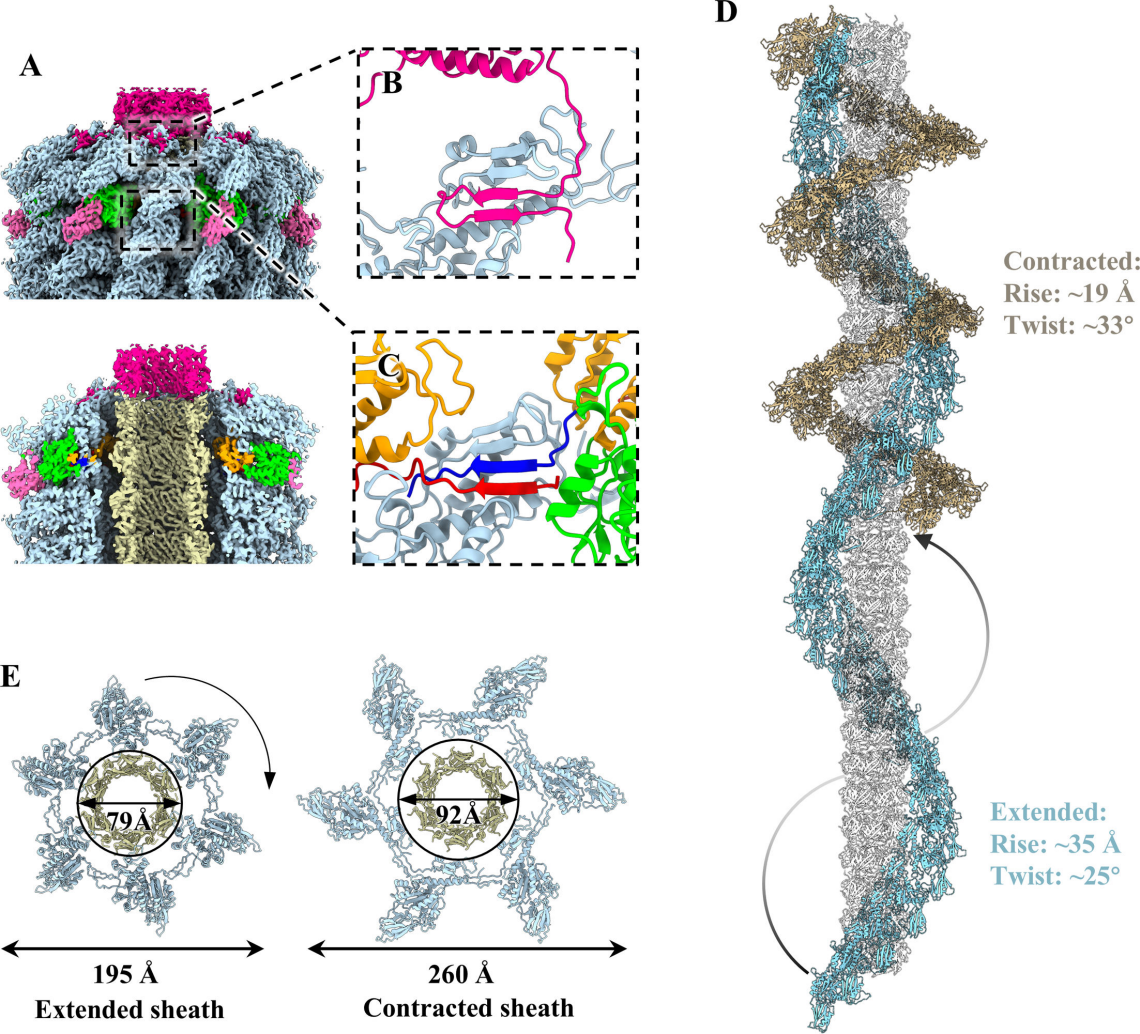
Structural changes of Mu in the extended and contracted states. (**A**) Side and cut-open views of the density maps of the tail terminator and tail in the contracted Mu. The color coding is identical to that used in Fig. 3A. (**B**) Zoomed-in view of the interactions between the domain I of a gp39 monomer in the final layer of tail and the C-terminal β-hairpin domain of a gp37 monomer in contracted Mu. (**C**) Zoomed-in view of the inter-ring and intra-ring interactions among sheath monomers in contracted Mu. (**D**) Ribbon models of all sheath monomers in one helical pitch of extended (light blue) sheaths versus contracted (goldenrod) sheaths along all tube rings (gray). (**E**) Top views of the conformational changes of the tail from the extended sheath to the contracted sheath.

The release of the TMP and DNA in contracted Mu results in a contracted tail and an empty head (Fig. 6A; Fig. S3A). However, in both extended and contracted states, the head and the connector complex remain unchanged (Fig. 1A and D; Fig. S16). The terminator protein gp37 remains unaltered in both states, with the exception of the C-terminal β-hairpin domain (Fig. S16). Upon contraction, the flexible C-terminal β-hairpin domain of gp37 elongates in conjunction with the domain I of the last layer of the tail sheath protein gp39, thereby accommodating sheath expansion (Fig. 3F and 6B).

In the tail, the tube protein gp40 does not undergo any conformational change. However, the sheath protein gp39 undergoes significant conformational changes in both states (Fig. S16). A structural comparison of the extended and contracted sheath monomers reveals that the N- and C-terminal arms of gp39 undergo significant conformational changes (Fig. S16), indicating that each gp39 monomer primarily undergoes a rigid-body rotation, mediated by its N- and C-terminal arms, upon contraction. The contracted sheath retains the four-stranded β-sheet arrangement of all gp39 monomers (Fig. 3E and 6C), but the helical rise decreases from approximately 35 Å to approximately 19 Å, and the helical twist increases from approximately 25° to approximately 33° (Fig. 6D). The outer diameter of the sheath ring increases from 195 Å to 260 Å, and the inner pore diameter expands from 79 Å to 92 Å, which is sufficient to break the contacts between the tail sheath and tube (Fig. 6E). This process enables the sheath to be detached from the tube, resulting in the sheath becoming more compact and contracting, and the tail tube becoming exposed (Fig. 6D and E). Notably, conformational changes of the sheath contraction in Mu display topologically identical to that of myophages E217 (14), P1 (57), and CIS R2 pyocin (4), where their sheaths usually exhibit compaction and contraction during contraction. As with previously reported CISs (8, 59, 63) and myophages (11, 14, 15), which transition from a high energy state to a low energy state during contraction, it is postulated that phage Mu releases the stored energy through sheath contraction, thereby providing sufficient force to penetrate the host cell membrane. Although the baseplate of the Mu is detached in the contracted state due to urea-treatment-induced reasons, a series of studies have demonstrated that contraction is triggered at the baseplate and propagates in a wave-like manner along the tail to the head (11, 66, 67).

## DISCUSSION

In this study, we determined the structures of Mu in both extended and contracted states at near-atomic resolutions and built the atomic models for almost all proteins from the head to the tail in extended Mu, as well as for the connector and tail in contracted Mu. The comparative analysis of the structural characteristics among Mu, other myophages (13, 16, 17), and CISs (4, 8, 63) reveals that the structure of the majority of connector-tail components of phage Mu is conservative. This indicates that Mu displays the essential and conserved components of a minimal myophage, suggesting that these are the minimum requirements for the construction of a functional and effective contractile nanomachine. In comparison to the structures of the extended and contracted Mu, the head, connector complex, and the tube of Mu exhibit minimal change, with the exception of the C-terminus of the terminator gp37, which extends to accommodate sheath contraction. In contrast, the sheath undergoes significant conformational changes, resulting in the compaction and contraction of the sheath and the exposure of the tube.

In particular, the BH1a proteins in the simple baseplates serve as a “glue” to affix the wedge to the hub (13, 14), and they often exhibit conserved structural characteristics (Fig. S10B). During the infection process, the wedge and BH1a in the baseplate undergo a reorientation and dissociation, respectively, resulting in the splaying out of the wedge and the release of the BH1a protein from the baseplate, both of which contribute to the tail contraction (13, 14). It is therefore hypothesized that the BH1a protein, which acts as a significant barrier, controls sheath contraction. It is noteworthy that the helix-rich domain of the tube initiator protein in phages Mu and Milano (16) is postulated to replace some of the functions of the BH1a protein, thereby playing a pivotal role in initiating tail contraction. Given the structural stability of the central region in the simple baseplate, it seems reasonable to posit that the Mu tube initiator protein remains attached to the baseplate. At present, there is no evidence to suggest that the distal tail in myophages is capable of penetrating the inner membrane or that the fusion of the distal tail with the inner membrane forms a transient hole, thereby providing a channel for DNA ejection into the host cytoplasm (14, 66). The sole exception is T4, where sheath contraction of T4 leads to the piercing of the outer membrane into the periplasm by the baseplate, and its TMP is postulated to cross the inner membrane (9). Prior experiments have demonstrated that the tube initiator protein gp43 in the Mu baseplate acts as a DNA cyclization protein, which is responsible for converting the linear form of phage DNA into a circular form (19, 28 ). This also indicates that gp43 is essential for the process of entering the host cytoplasm. There is indirect evidence to suggest that the Mu distal tail is capable of spanning the host cell envelope and entering the host cytoplasm. These findings suggest that each myophage may have its own strategy for crossing the host cell envelope. In general, in comparison with the reported BH1a proteins or tube initiator proteins among myophages, the tube initiator protein gp43 of Mu is a multifunctional protein that plays a pivotal role in the baseplate assembly, tube assembly, baseplate stability, tail contraction, and DNA cyclization.

The Mu baseplate in the extended state displays structural similarity to those in myophage E217 (14) and CIS R2 pyocin (4), all of which include both extended and contracted states with the baseplate. Consequently, by analogy, we have been able to propose a molecular mechanism for tail contraction of phage Mu (Fig. S17). In the initial stage, the fibers move freely up and down, searching for the receptor LPS on the outer membrane of *E. coli* and transmitting the signal to the wedge (Fig. S17A). As previously reported for myophage E217 (14) and R2 pyocin (4), all wedges may be splayed out, thereby initiating conformational changes of the entire baseplate (Fig. S17B and C). The peripheral sheath next contracts toward the neck and moves upwards as a wave along the tail tube (Fig. S17D), whereas the head-connector-tube rotates in a coordinated direction (Movie S1). The energy released by the contraction of the tail sheath facilitates the penetration of the distal tail into the host cell envelope, enabling access to the host cytoplasm. Subsequently, the Mu DNA is released into the host cytoplasm. The tube initiator protein gp43 then mediates the cyclization of the Mu DNA, thereby preventing it from being cleaved (Fig. S17E). Ultimately, Mu DNA is integrated into the host’s DNA, thereby initiating the lysogenic cycle or lytic cycle (Fig. S17F) (68). The structures of Mu in extended and contracted states provide a structural basis for understanding the assembly pattern and contraction mechanism among myophages and CISs. Our study offers a framework to support a more comprehensive understanding of phages as potential biomedicines for phage therapy applications and genetic tools and inform engineering opportunities.

## MATERIALS AND METHODS

### Production and purification of phage Mu

*E. coli* strain (ATCC 23724) was cultivated in LB Broth medium for 24 h at 37°C. The recovered *E. coli* strain was then re-cultured at 37°C for 4 h. Mu phage was inoculated into logarithmic growth *E. coli* cells for 8 h at 37°C. The Mu phage culture was lysed by the addition of a small quantity of chloroform. Subsequently, the cell debris was removed by low-speed centrifugation at 6,000 g for 20 min at 8°C. The supernatant was enriched by 10% polyethylene glycol 8000 and 1 M NaCl precipitation overnight at 4°C. The precipitated phage particles were resuspended in phage buffer (10 mM MgCl_2_, 5 mM NaCl, and 10 mM Tris-HCl, pH 7.4), and subsequently purified by CsCl gradient centrifugation (1.5 g/mL and 1.4 g/mL). The complete ultracentrifugation process was performed at 135,000 g for 2 h at 8°C. Subsequently, the Mu phage bands were dialyzed in phage buffer at 4°C overnight.

By referring to the processing method of contracted T4 (69), the Mu phage particles with contracted tails were treated as follows: the Mu phage sample was diluted 10-fold with the addition of 5 M urea and then incubated in phage buffer for 2 h at 37°C. The contraction of the phage tail was observed using negative staining and cryo-EM. Urea was removed by ultrafiltration, and the sample concentration was increased to facilitate the final cryo-EM preparation. Finally, both extended and contracted states of phage Mu were collected and stored in ice water for cryo-sample processing.

### Data acquisition and icosahedral reconstruction

Three microliters of native phage and urea-treated phage were applied to a Quantifoil R2/1 copper grid, which had been glow-discharged for 30 s. The grids were loaded into an FEI Vitrobot with 8°C, 100% humidity, and a blot time of 4.0 s. The grids were then plunged frozen into a solid-liquid ethane and stored in liquid nitrogen. The cryo-EM data were recorded using a 300 kV Titan Krios G3i electron microscope, equipped with a K3 summit direct electron detector. The FEI EPU software application proceeded to automatically collect data of native phage and urea-treated phage at a magnification of 53,000× and 64,000×, resulting in a pixel size of 1.36 Å and 1.06 Å, respectively. The full dose of each movie of native phage and urea-treated phage was approximately 35 e^−^/Å^2^. A total of 3,353 movies were collected for the native phage, and 5,770 movies for the urea-treated phage. The movie stacks were drift-corrected and dose-weighted using MotionCor2 (70), with the first and last frames excluded, thereby generating summed images with dose weighting. The astigmatism and defocus values of each image were determined using Gctf software (71). A total of 55,035 and 42,000 particle images of the extended Mu and the contracted Mu, respectively, were selected for analysis using the software ETHAN (72). The icosahedral reconstruction of the extended Mu particles was performed using our own software (37), which is based on the common-line algorithm (73, 74). To further improve the resolution of the head, the local reconstruction method (38) was employed to reconstruct the 3-fold capsid of the icosahedral head to a resolution of 3.2 Å.

### Symmetry-mismatch and local reconstruction

The asymmetric structure of myophage Pam3 (13), filtered to 60 Å resolution, was used as the initial model. The 3D asymmetric structure of the head-connector complex in the extended Mu was reconstructed at a low resolution by using the symmetry-mismatch reconstruction method (39, 40). The reconstruction process is outlined as follows: (1) For each two-dimensional (2D) particle image, the asymmetrical orientations were searched from the 60 equivalent orientations of the icosahedral orientation based on an initial model (2). The latest orientations were then employed in the reconstruction of a new asymmetric structure, without any imposition of symmetry (3). The aforementioned steps were repeated until the orientations of all particle images remained unchanged. Subsequently, the portal-adaptor and the terminator-tail region were improved to resolutions of 3.4 Å and 3.5 Å, respectively, by imposing C12-fold and C6-fold symmetries. By employing the same methodology, the structures of the portal-adaptor and the terminator-tail region of the contracted Mu were reconstructed at resolutions of 3.6 Å and 3.8 Å, respectively.

The local reconstruction of the baseplate of the extended Mu particles was performed using the RELION 3.1.1 software (41). A total of 55,000 baseplate particles were manually picked with a box size of 300 × 300 pixels. Subsequently, 2D and 3D classifications were performed. After the removal of the structure was deemed irrelevant, 46,000 particles were selected for 3D refinement to a resolution of 4.2 Å, imposing 6-fold symmetry. To further improve the resolution of the 3-fold symmetric structure, the particle orientation in the orientation file was expanded by the imposition of 6-fold symmetry, using the “relion_particle_symmetry_expand” command. Furthermore, each 2D particle image was corrected again using the CTF refinement process. Thereafter, all particle images were subjected to direct 3D refinement to a resolution of 3.3 Å with 3-fold symmetry imposed. The flow charts for the 3D reconstruction of the baseplate are presented in Fig. S18.

### Atomic modeling building and refinement

Based on the cryo-EM density maps of the extended Mu, we manually built models for MCP gp34, portal protein gp29, adaptor protein gp36, terminator protein gp37, sheath protein gp39, tube protein gp40, the C-terminus of TMP gp42, tube initiator protein gp43, hub protein gp44, spike protein gp45, BW1 gp46, BW2 gp47, BW3 gp48, and the N-terminus of fiber gp49 by using COOT software (75). Based on density maps of the contracted Mu, we built models for portal protein gp29, adaptor protein gp36, terminator protein gp37, sheath protein gp39, and tube protein gp40. All models were refined using the real-space refinement method, implemented in Phenix (76). The final models were evaluated using the Ramachandran plots and MolProbity software (77). The refinement and validation statistics are presented in Table S1.

## Data Availability

Cryo-EM maps and the associated structural coordinates have been deposited into the Electron Microscopy Data Bank (EMDB) and the Protein Data Bank (PDB), respectively, under the following accession codes: EMD-61659, EMD-62358, EMD-62359, EMD-62362, EMD-62462, EMD-63137, 9JOD, 9KHX, 9KHY, 9KI1, 9KNU, and 9LJ8.

## SUPPLEMENTAL MATERIAL

Supplemental material jvi.02056-24-s0001.pdf Figures S1 to S18; Tables S1 and S2.

Movie S1 jvi.02056-24-s0002.mp4 Dynamic diagram of Mu from extended to contracted.

ASM does not own the copyrights to Supplemental Material that may be linked to, or accessed through, an article. The authors have granted ASM a non-exclusive, world-wide license to publish the Supplemental Material files. Please contact the corresponding author directly for reuse.
