# Supplementary material for: In situ structures of the contractile nanomachine myophage Mu in both its extended and contracted states
Source: J Virol. 2025 Feb 24;99(3):e02056-24. doi: 10.1128/jvi.02056-24 (PMC11915804; doi:10.1128/jvi.02056-24)
Supplement: Supplemental material — Figures S1 to S18; Tables S1 and S2. [file jvi.02056-24-s0001.pdf]

Supplementary information for

# In–situ structures of the contractile nanomachine Myophage Mu in both its extended and contracted states

Junquan Zhou[1,#], Liwen Wang[1,#], Hao Xiao[1], Wenyuan Chen[1], Zhonghua Liu[2], Jingdong Song[3], Jing Zheng[1,2,*], Hongrong Liu[1,*]

This PDF file includes:

Supplementary Figures S1 to S18

Supplementary Tables S1 to S2

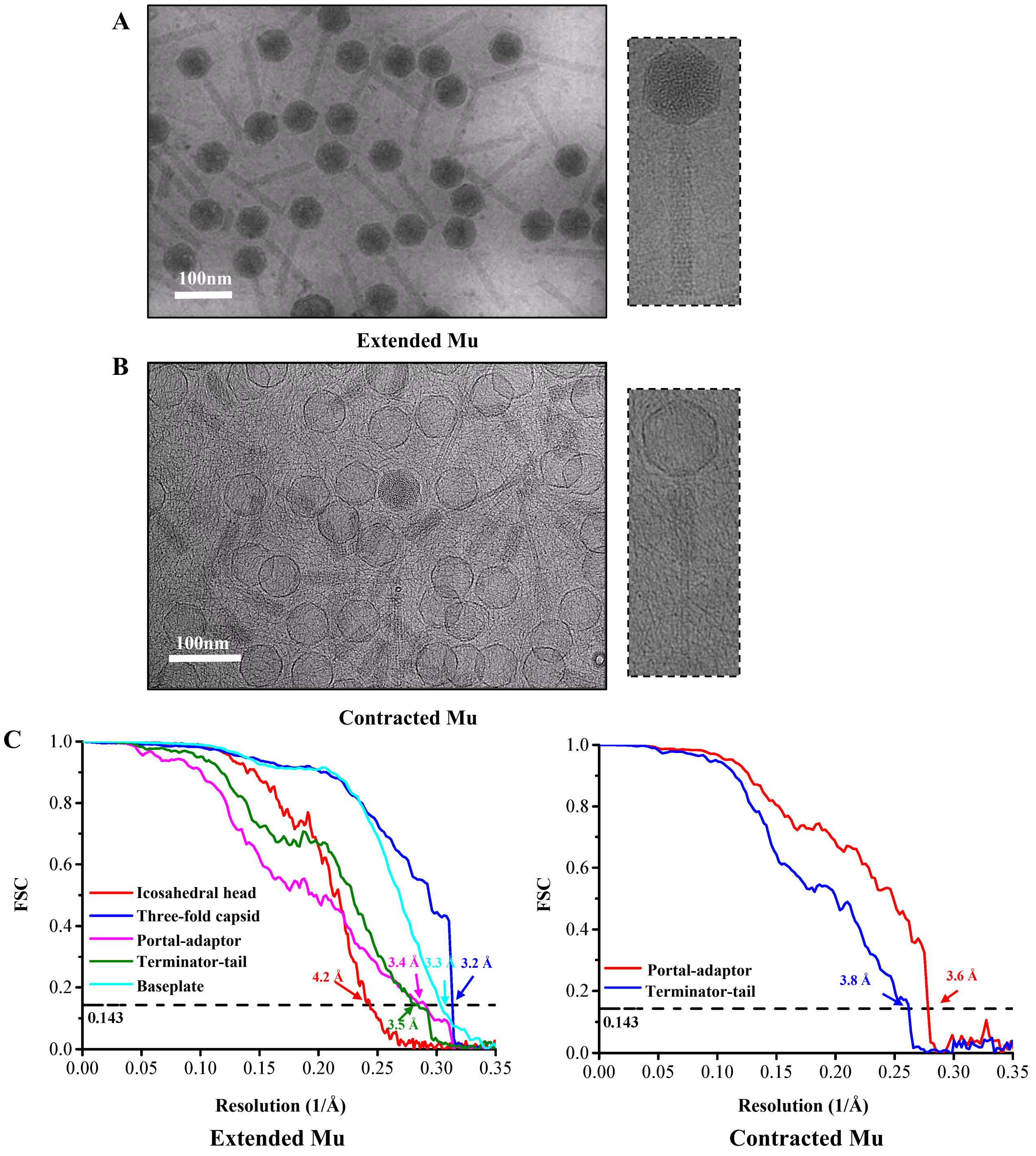


**Figure S1. Cryo-EM images and Fourier shell correlation curves of phage Mu in the extended and contracted states. (A, B)** Representative cryo-EM images (left) and zoomed-in view (right) of extended (A) and contracted Mu particles (B). **(C)** Left: estimated structural resolutions of the icosahedral head (red line), the three-fold capsid of the icosahedral head (blue line), the portal-adaptor (pink line), the terminator-tail region (green line), and the baseplate (cyan line) in extended Mu. Right: estimated structural resolutions of the portal-adaptor (red line), and the terminator-tail region (blue line) in contracted Mu.

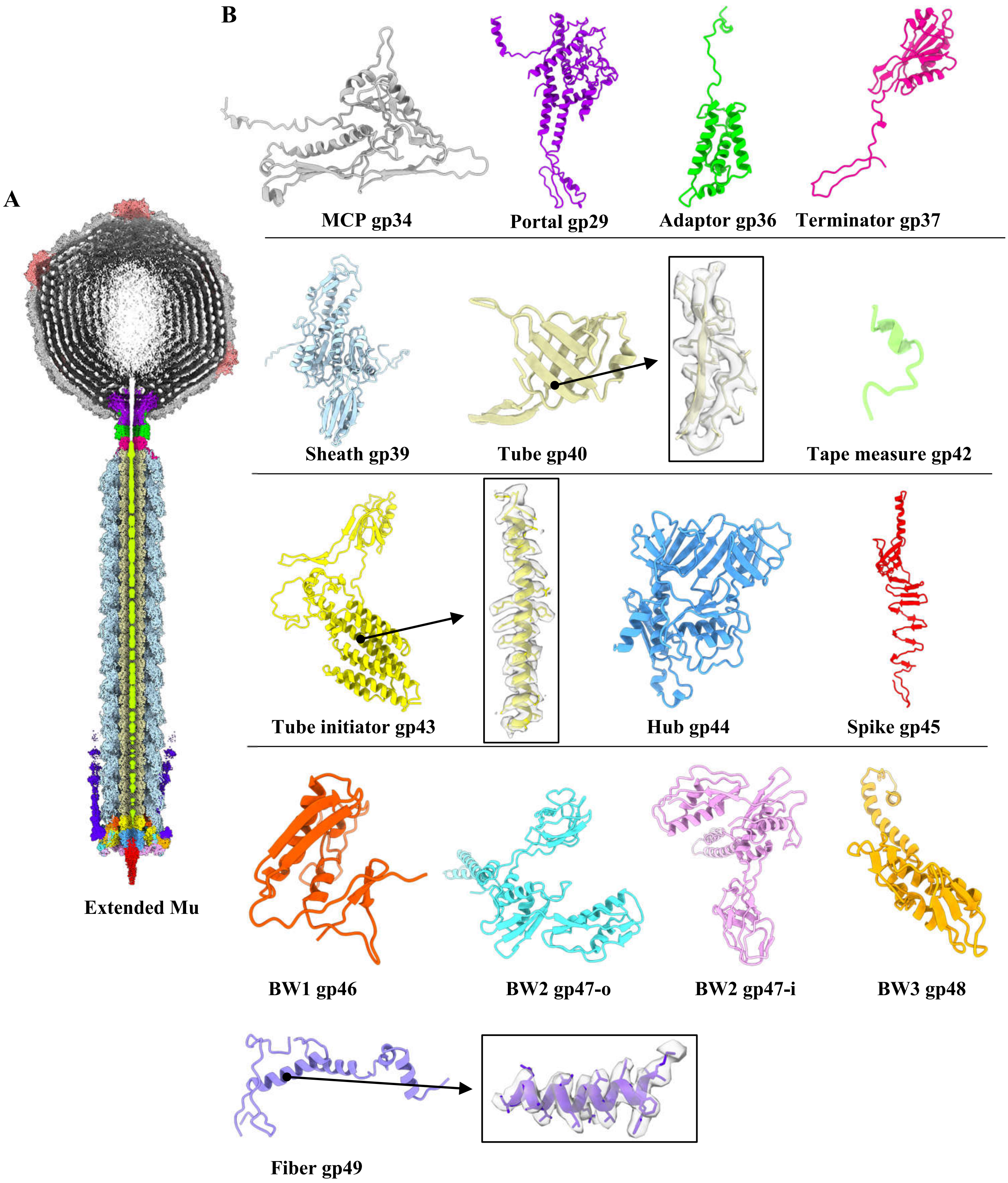


**Figure S2. Quality of the cryo-EM density maps and the atomic models from the head to the tail in extended Mu**. **(A)** Cut-open view of the asymmetric structure of extended Mu. **(B)** Ribbon models of all protein components from the head to the tail and zoomed-in views of density maps (transparency) superimposed on the atomic models of partial proteins (sticks). All color codes are identical to that used in Fig. 1.

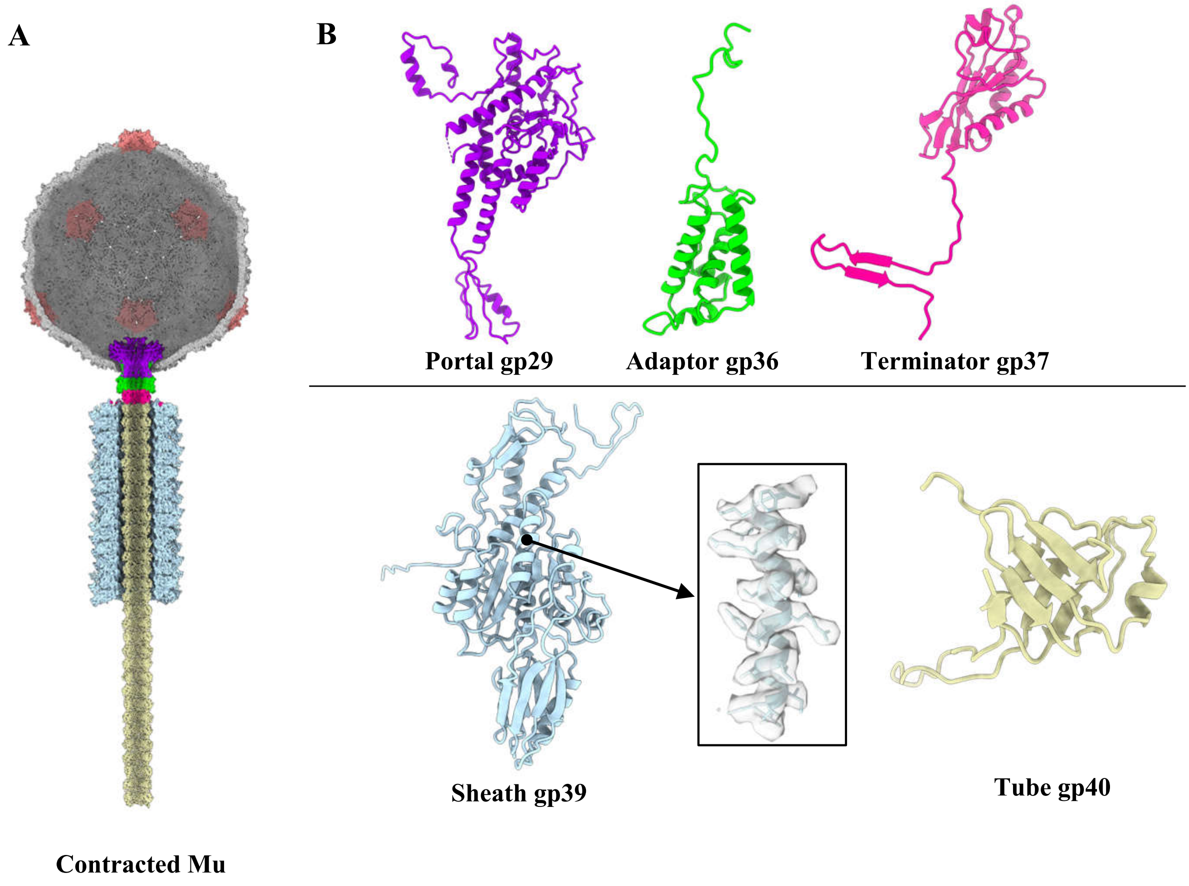


**Figure S3. Quality of the cryo-EM density maps and the atomic models of the connector-tail region in contracted Mu**. **(A)** Cut-open view of the asymmetric structure of contracted Mu. **(B)** Ribbon models of the connector-tail proteins and zoomed-in view of density map (transparency) superimposed on atomic model of gp39 (sticks). All color codes are identical to that used in Fig. 1.

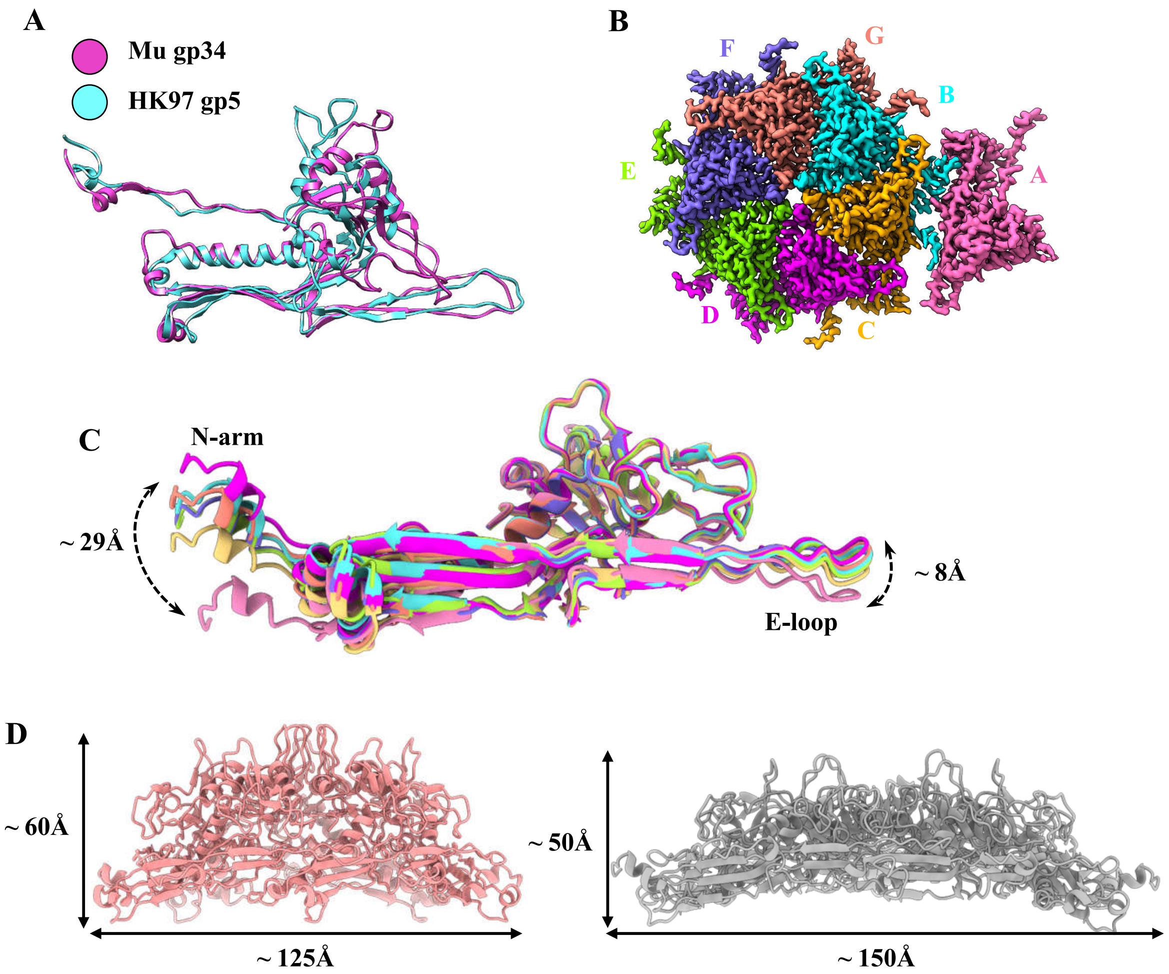


**Figure S4. Structure of the MCP gp34 of Mu. (A)** Structural comparison of the ribbon models between the Mu MCP gp34 and the HK97 MCP gp5 (PDB ID: 1OHG). **(B)** Density maps of the seven MCP monomers (labeled A-G) in an asymmetric unit of Mu head. **(C)** Superimposition of the seven MCPs in an asymmetric unit. The color codes are identical to that used in Fig. S4B. **(D)** Side view of the ribbon models of the penton (left, salmon) and the hexon (right, grey).

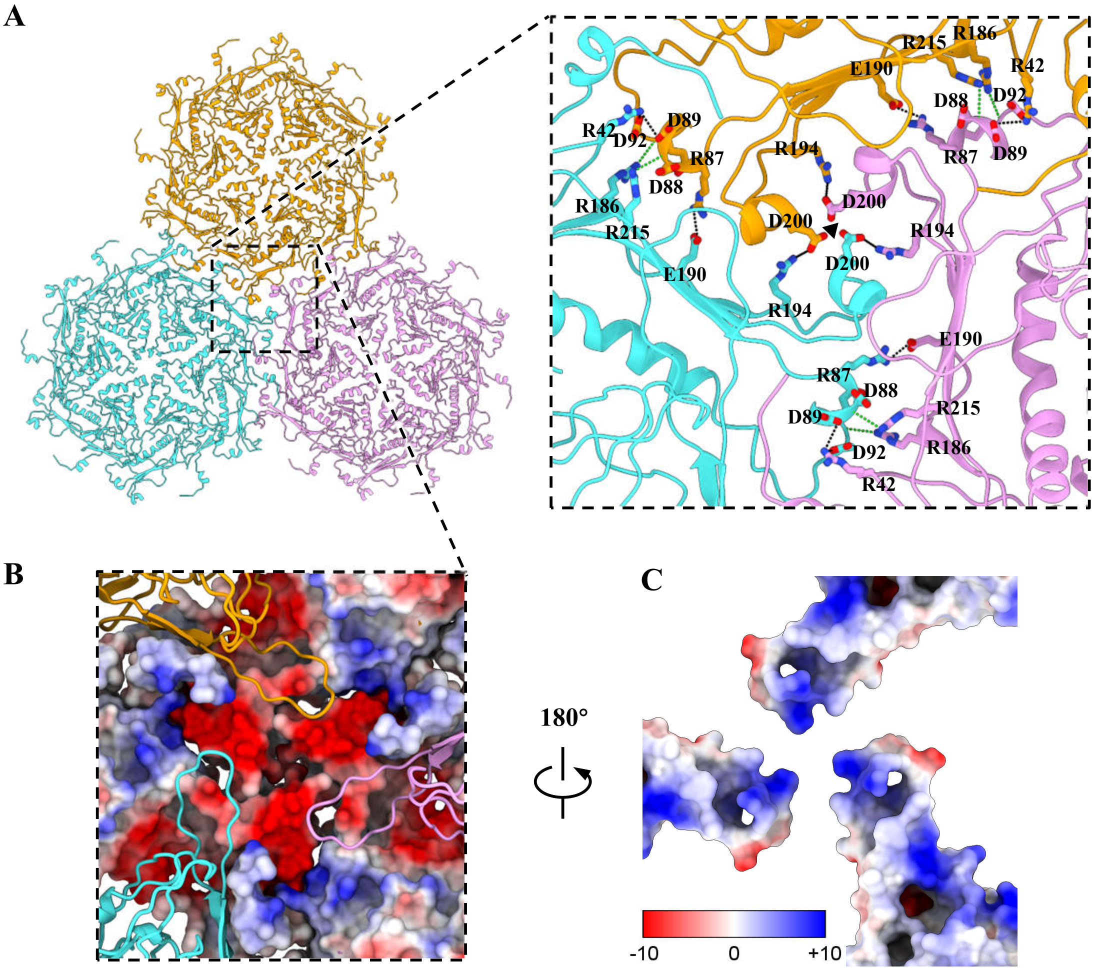


**Figure S5. Interactions at the capsomere-capsomere interface around the three-fold axis and quasi-three-fold axis of the icosahedral head. (A)** Top view of the interactions along the three-fold axis and zoomed-in view of the inter-capsomeric interactions. The three hexons are colored cyan, orange, and pale pink, respectively. H-bonds and salt bridges are indicated by dashed green lines and dashed black lines, respectively. **(B)** Top view of the electrostatic potential surface around the three-fold axis, with the exception of the E-loops and partial A-domains, which are illustrated in ribbon models. **(C)** Bottom view of the electrostatic potential surface of E-loops and partial A-domains coming from the panel B. The electrostatic potential scale is shown in the color bar.

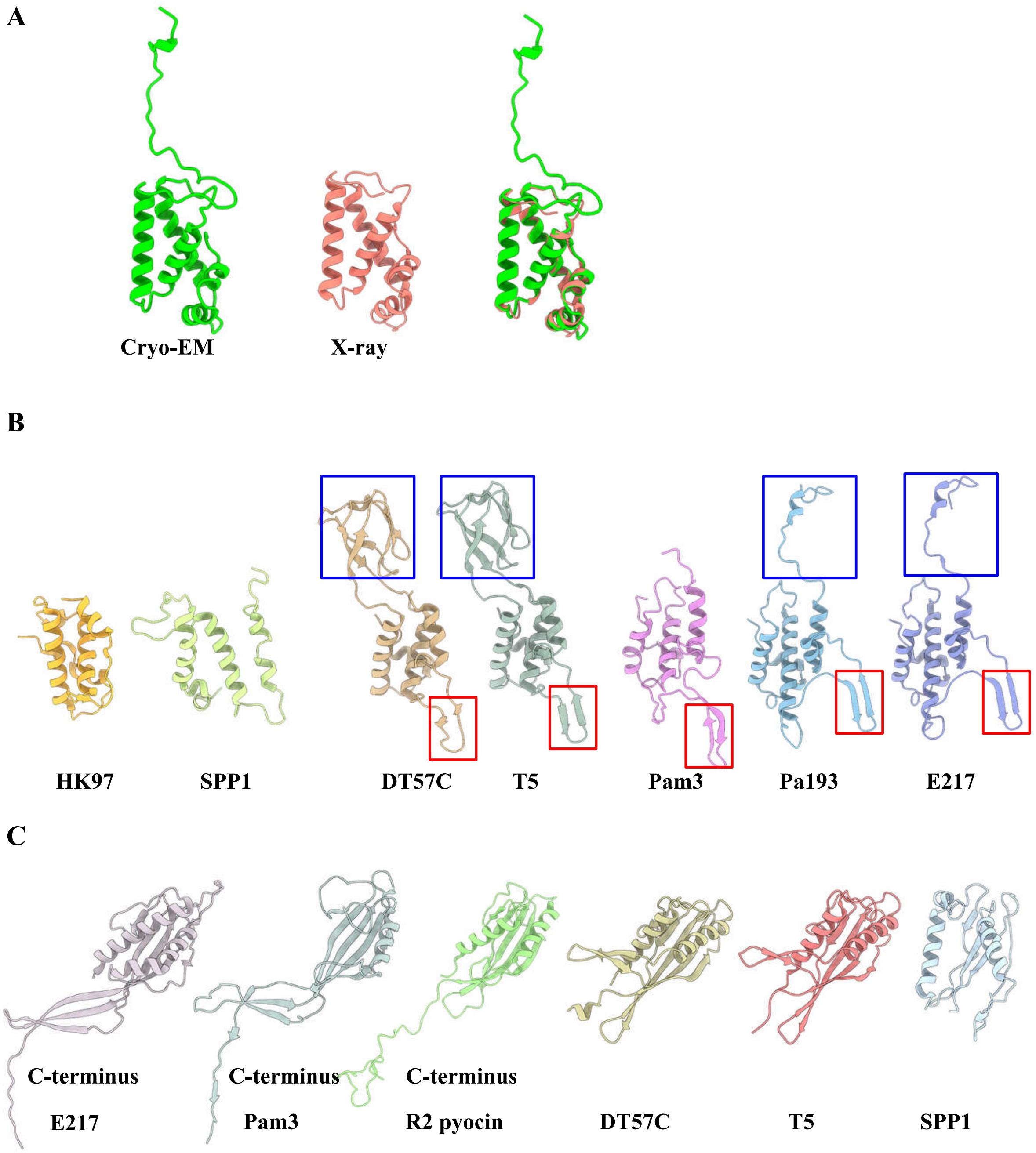


**Figure S6. Structural comparisons of the connector proteins of Mu, other myophages, and CISs. (A)** Structural comparison of adaptor protein gp36 in our structure (green) and the X-ray crystal structure (salmon, PDB ID: 5YDN). **(B)** Structural comparisons of the adaptor proteins among siphophages and myophages, including HK97 (PDB ID: 3JVO), SPP1 (PDB ID: 5A20), DT57C (PDB ID: 8HQO), T5 (PDB ID: 9ILP), Pam3 (PDB ID: 8HDS), Pa193 (PDB ID: 9B41) and E217 (PDB ID: 8FVH). The β-hairpin structure and C-terminus are marked with red rectangular boxes and blue rectangular boxes, respectively. The root-mean-square deviation (RMSD) value of Cα atoms of the adaptor proteins between Mu and T5, DT57C, HK97, Pam3, Pa193, E217 is 3.4 Å, 3.4 Å, 2.8 Å, 3.7 Å, 3.8 Å, 4.2 Å, respectively. **(C)** Structural comparisons of the terminator proteins among myophages, CISs and siphophages, including R2 pyocin (PDB ID: 6U5F), E217 (PDB ID: 8FVH), Pam3 (PDB ID: 8HDR), DT57C (PDB ID: 8HQO), T5 (PDB ID: 9ILV), SPP1 (PDB ID: 5A20). The RMSD value of Cα atoms of the terminator proteins between Mu and E217, Pam3, R2 pyocin, DT57C, T5, SPP1 is 3.9 Å, 4.4 Å, 3.7 Å, 3.0 Å, 3.1 Å, 6.8 Å, respectively.

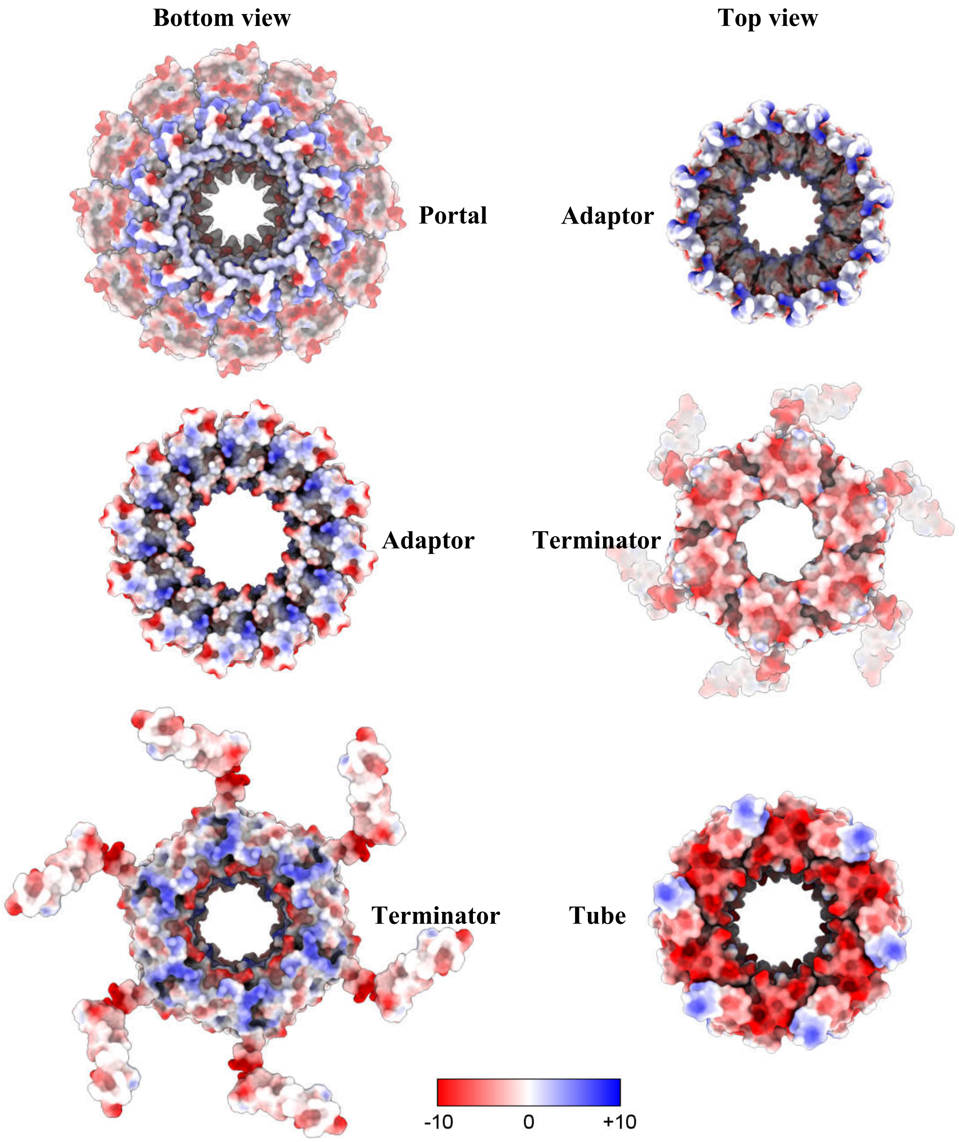


**Figure S7. Electrostatic potential surfaces of the interacting regions between two adjacent protein components in extended Mu.** The left columns are oriented toward the baseplate, whereas the right columns are oriented toward the head. The electrostatic potential scale is shown in the color bar.

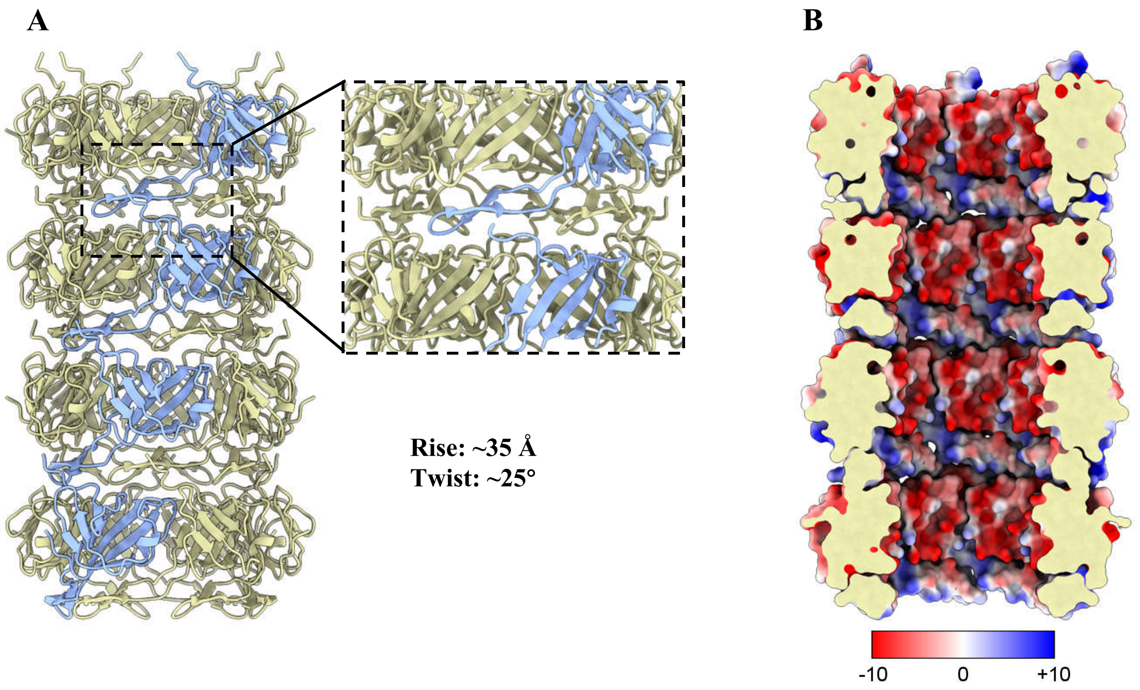


**Figure S8. Structure of the tail tube of extended Mu. (A)** Side view of the four layers of tail tube and zoomed-in view of the inter-ring interactions by each gp40 monomer. **(B)** Cut-open view of electrostatic potential surfaces of the inner surfaces in tail tube. The electrostatic potential scale is shown in the color bar.

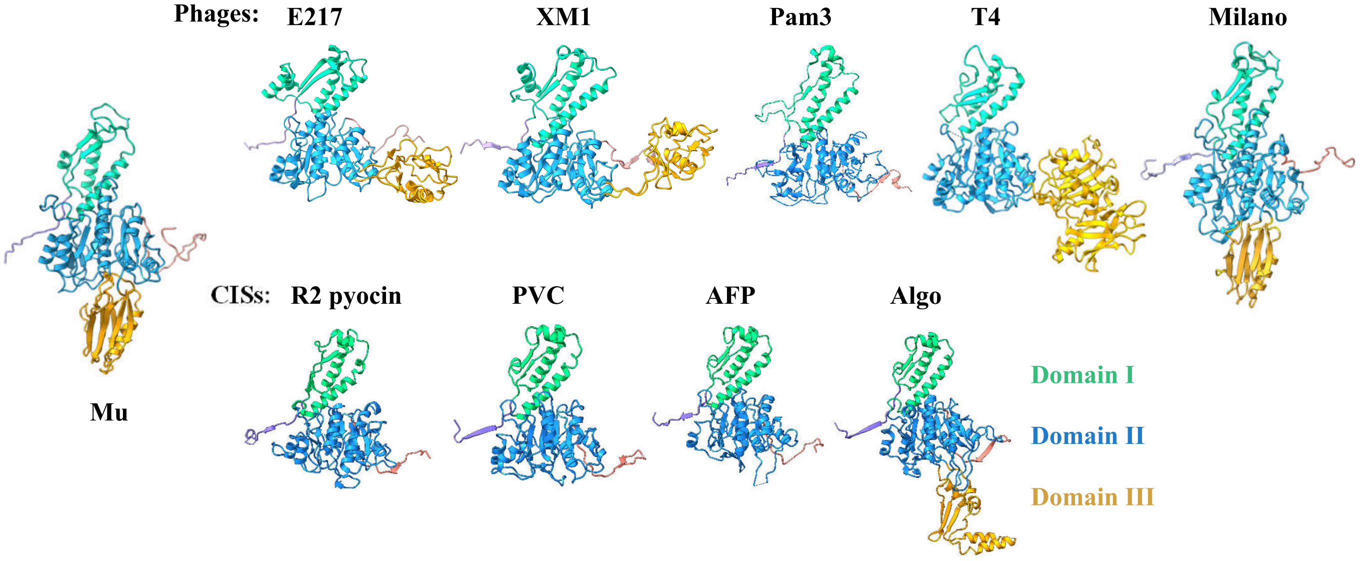


**Figure S9. Structural comparisons of the tail sheath proteins of Mu, other myophages, and CISs**. Phages E217 (PDB ID: 8ENV), XM1 (PDB ID: 7KH1), Pam3 (PDB ID: 8HDW), T4 (PDB ID: 3J2N) and Milano (PDB ID: 8FQC), as well as CISs R2 pyocin (PDB ID: 3J9Q), PVC (PDB ID: 6J0F), AFP (PDB ID: 6RBN) and Algo (PDB ID: 7AE0) are colored according to their domains.

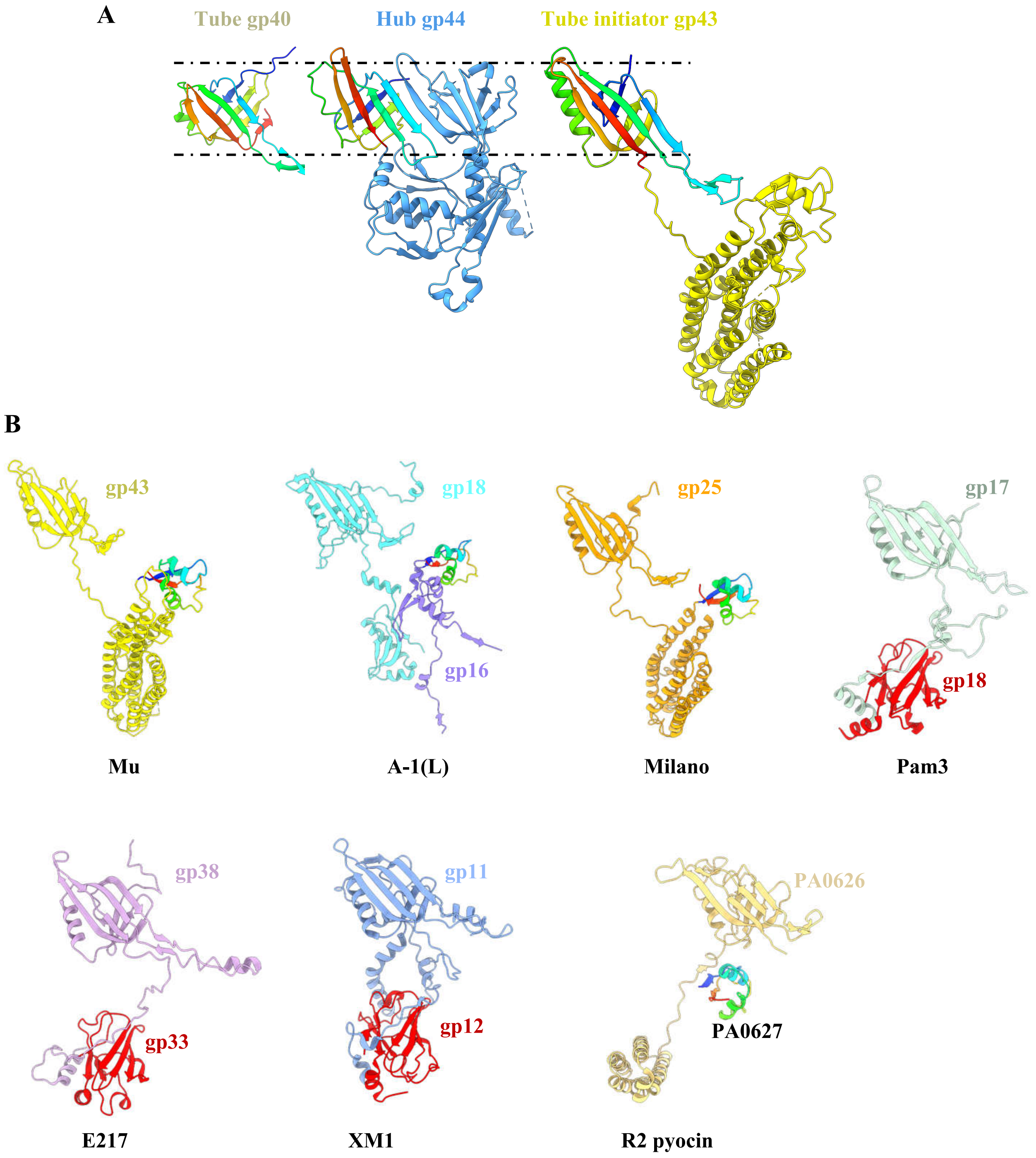


**Figure S10. Structural comparisons of the tube initiator proteins. (A)** Structural comparisons of the tube-like domain (rainbow colors) among proteins gp43, gp40 and gp44 in extended Mu. **(B)** Structural comparisons of the ribbon models of the tube initiator protein among myophages and CISs, including Mu, A-1(L) (PDB ID: 8KEA), Milano (PDB ID: 8FQC), Pam3 (PDB ID: 7YFZ), E217 (PDB ID: 8EON), XM1 (PDB ID: 7KH1) and R2 pyocin (PDB ID: 6U5B). The LysM domain and BH1a protein are shown in rainbow colors and red, respectively, the location of gp16 (purple) in A-1(L) is similar to the BH1a protein.

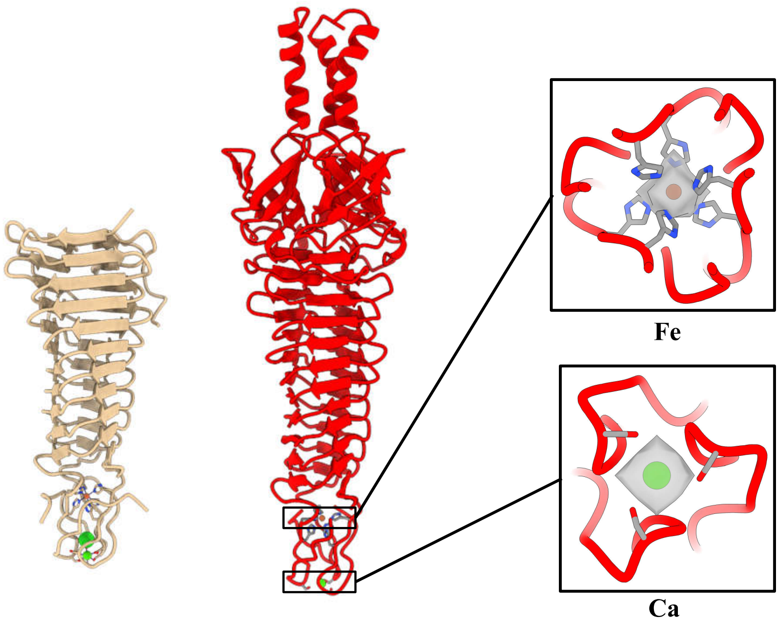


**Figure S11. Structural comparison of the trimeric spike gp45 in our structure (red) and the X-ray crystal structure (tan, PDB ID: 3VTO).** The insets show the zoomed-in views of density maps of Fe and Ca ions.

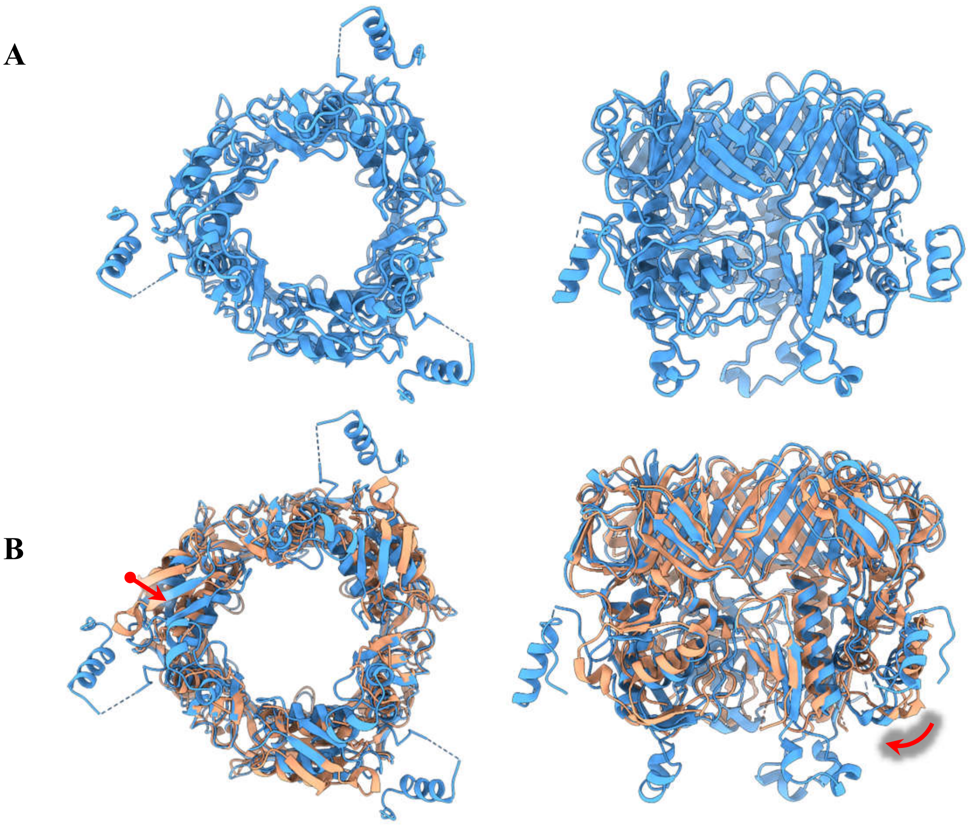


**Figure S12. Structure of the hub of extended Mu. (A)** Bottom (left) and side (right) views of the ribbon models of hub protein gp44. **(B)** Structural comparison of the hub in our structure (sky blue) and the X-ray crystal structure (coral, PDB ID:1WRU), red arrows indicate that the C-terminus of the hub of the in situ structure is shifted inward relative to the X-ray crystal structure.

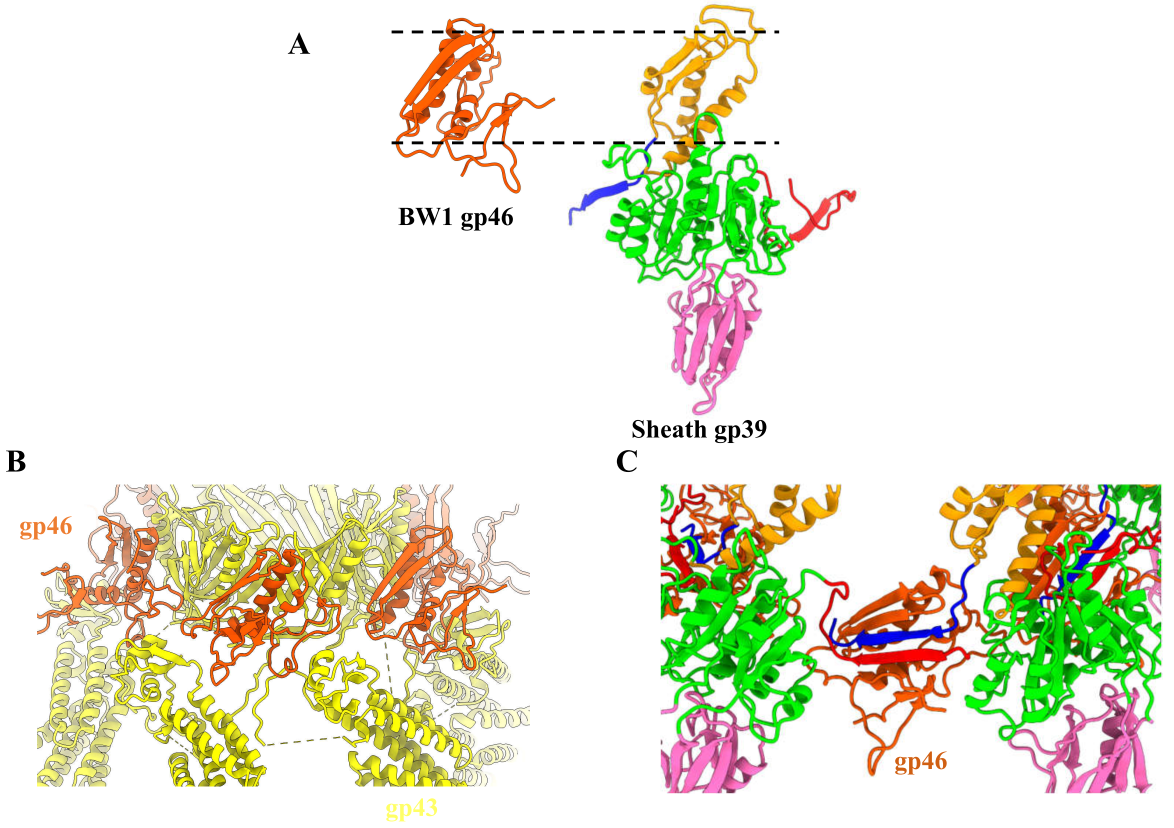


**Figure S13. Structure of the BW1 gp46 of extended Mu. (A)** Structural comparison of the ribbon models of the BW1 gp46 and the sheath protein gp39. **(B)** Zoomed-in view of the location of the BW1 protein gp46 around the tube initiator gp43. **(C)** Zoomed-in view of the interaction between a BW1 gp46 and two adjacent sheath proteins gp39 by forming a four-stranded β-sheet. The color code of sheath gp39 is identical to that used in Fig. 3C.

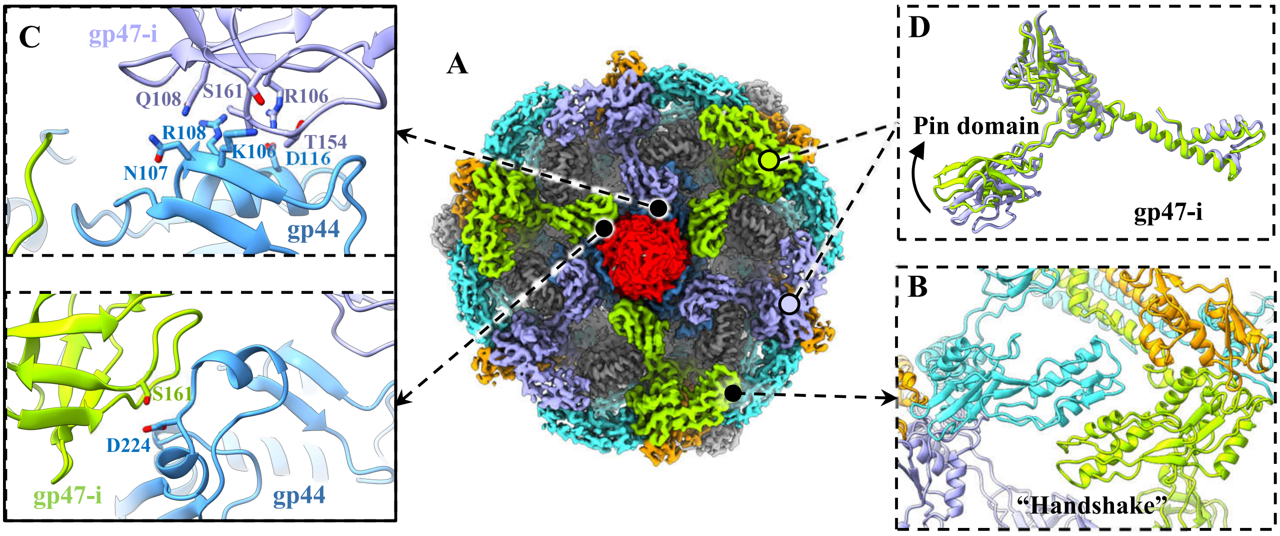


**Figure S14. Structure of the baseplate wedges of extended Mu. (A)** Bottom view of the density maps of baseplate. The color code is identical to that used in Fig. 1A, except gp47-i monomers are alternatively colored in lawn green and slate blue. **(B)** Zoomed-in view of a handshaking contact between gp47-i of a wedge and gp47-o of the adjacent wedge. **(C)** Zoomed-in views of the interactions between the pin domain of gp47-i and hub gp44. **(D)** Structural comparison of the ribbon models of two adjacent proteins gp47-i.

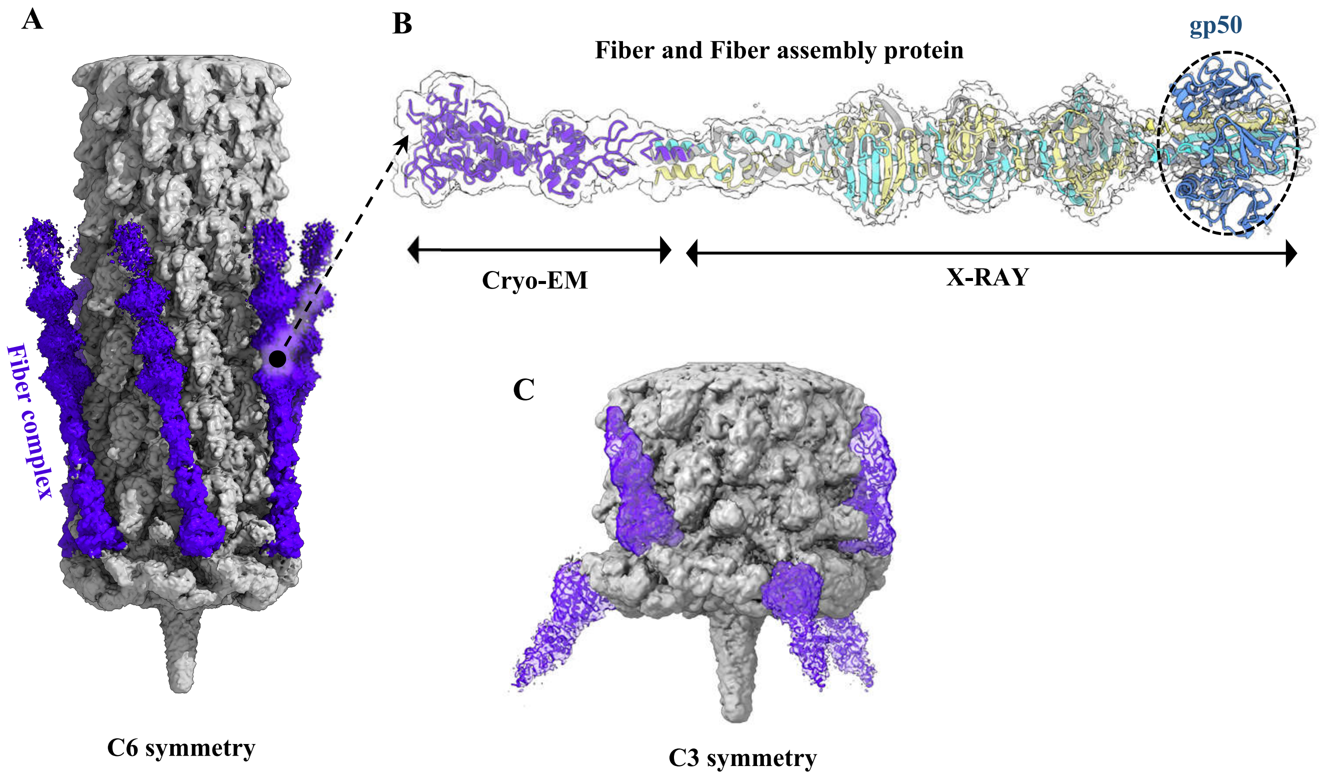


**Figure S15. Structure of the fiber of extended Mu. (A, C)** Side views of the density maps of the distal tail (gray) by imposing 6-fold symmetry (A) and 3-fold symmetry (C). All fibers are colored in purple. **(B)** Zoomed-in view of density map superimposed on fiber atomic models, including our N-terminus of gp49 (purple) and the reported X-ray crystal structure (PDB ID:5YVQ).

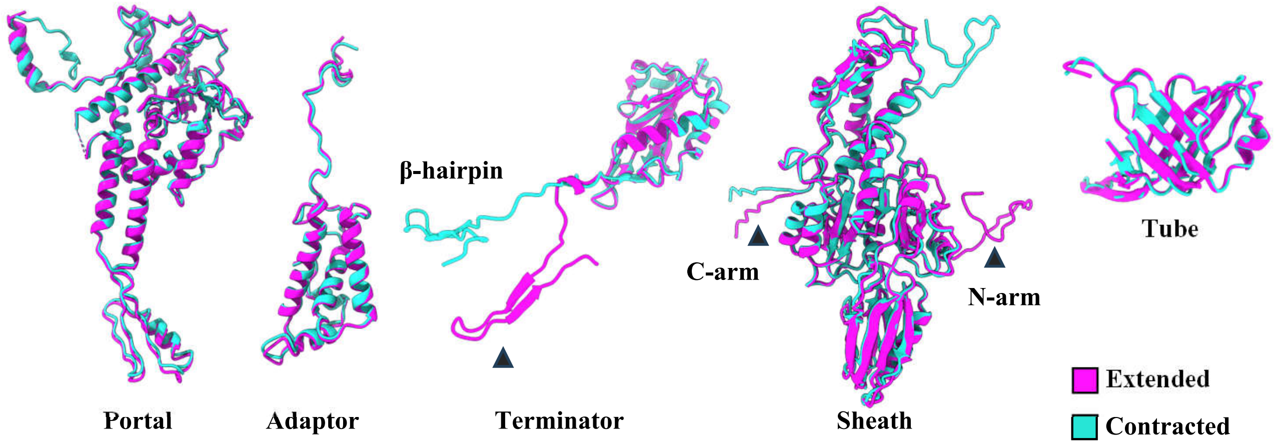


**Figure S16. Structural comparisons of the connector complex and the tail region in extended Mu and contracted Mu.**

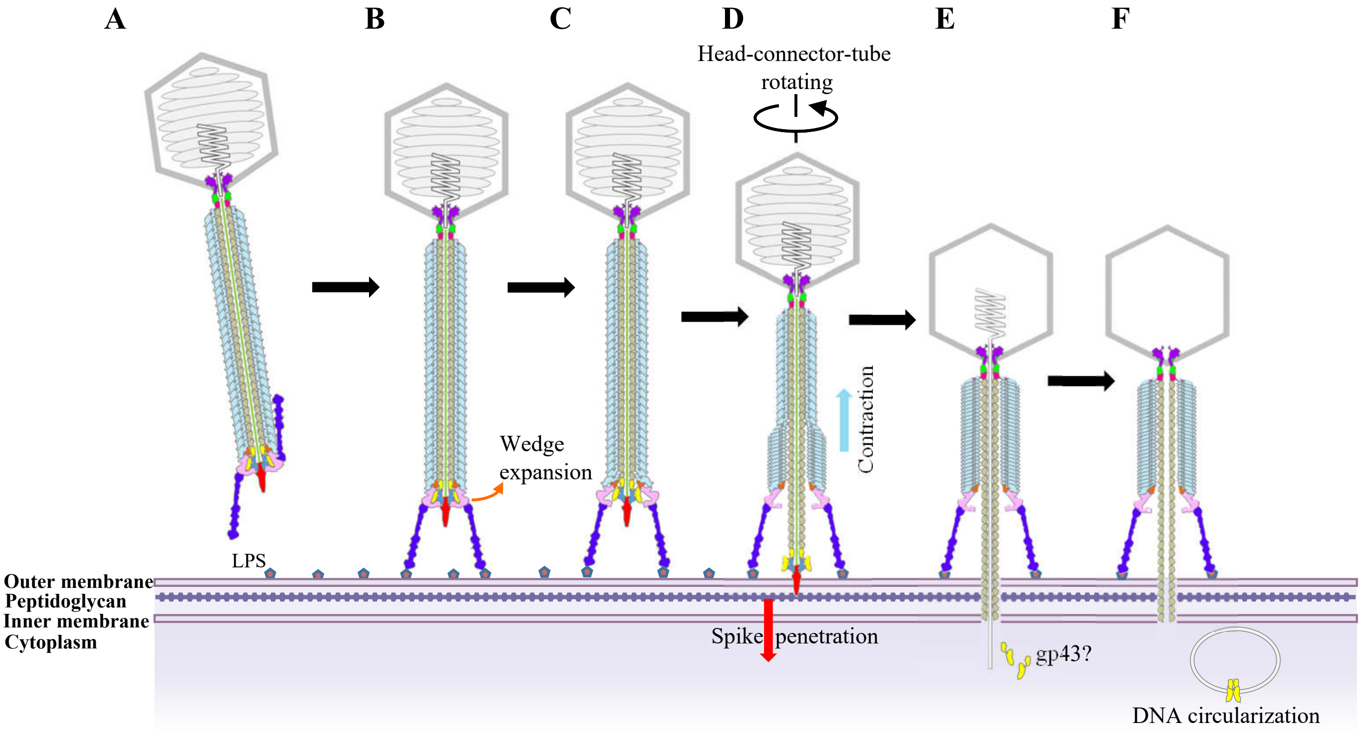


**Figure S17. Schematic diagram of Mu infection and DNA-ejection pathway. (A)** Phage Mu in extended state. **(B)** Fibers adsorb to host receptor LPS. **(C)** Wedges are dissociated from the baseplate, thereby initiating the contraction of the sheath. **(D)** The head-connector-tube rotates with contraction. **(E)** Distal tail then penetrates across the host cell envelope, facilitating the subsequent entry of the DNA into the host cytoplasm. **(F)** Tube initiator protein gp43 is responsible for the cyclization of Mu DNA. The color coding is identical to that used in Fig. 1.

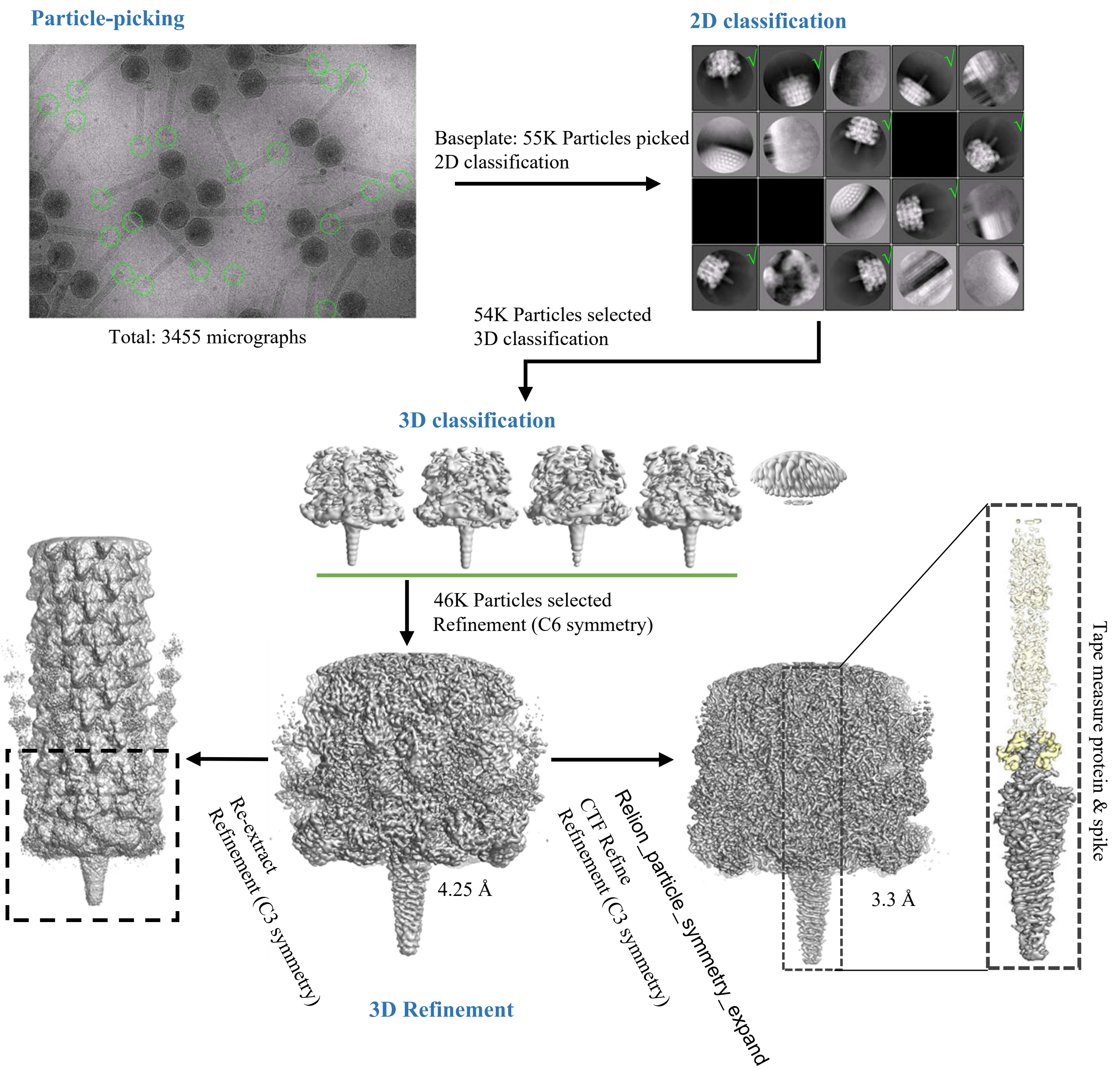


**Figure S18. Schematic diagram of cryo-EM data processing of baseplate segments in extended Mu.**

**Supplemental Table S1** Cryo-EM data statistics.

| DATA Collection | | |
|---|---|---|
| | Extended Mu | Contracted Mu |
| Electron microscopy | FEI 300kV Titan Krios G3i, Gantan K3 camera | |
| Pixel size (Å) | 1.36 | 1.06 |
| Total micrographs | 3353 | 5770 |
| Electron exposure (e/Å$^2$) | 30.5 (32 frames) | 37.5 (50 frames) |
| Voltage (KV) | 300 | |
| CS | 0 | 2.7 |
| Defocus range | -1.6 ~ 2.2 µm | |

| Map Refinement | | | | | | |
|---|---|---|---|---|---|---|
| | Extended Mu | | | | Contracted Mu | |
| Map | Three-fold capsid | Portal-Adaptor | Terminator-Tail | Baseplate (C3) | Portal-Adaptor | Terminator-Tail |
| Map resolution (Å) | 3.2 | 3.4 | 3.5 | 3.8 | 3.6 | 3.8 |
| Final particles | 60k | 55k | 67k | 45k | 42k | 42k |
| B-factor | 80 | 40 | 80 | 100 | 100 | 100 |
| EMDB-ID | EMD-61659 | EMD-62358 | EMD-62359 | EMD-62362 | EMD-62462 | EMD-63137 |

| Atomic Refinement | | | | | | |
|---|---|---|---|---|---|---|
| | Extended | | | | Contracted | |
| Protein | gp34 | gp29, gp36 | gp37, gp39, gp40 | gp42 - gp49 | gp29, gp36 | gp37, gp39, gp40 |
| PDBID | 9JOD | 9KHX | 9KHY | 9KI1 | 9KNU | 9LJ8 |
| **Ramachandran Plot** | | | | | | |
| Favored (%) | 96.32 | 98.84 | 97.32 | 95.46 | 97.67 | 94.97 |
| Allowed (%) | 3.68 | 1.16 | 2.68 | 4.54 | 2.33 | 5 |
| Disallowed (%) | 0 | 0 | 0 | 0 | 0 | 0.02 |
| MolProbity score | 1.45 | 1.32 | 1.42 | 1.71 | 1.49 | 1.93 |
| Clashscore | 4.2 | 5.8 | 5.42 | 6.93 | 7.98 | 11.79 |
| Poor rotamers (%) | 0.22 | 0 | 0 | 0.15 | 0 | 0.06 |

**Supplemental Table S2** Protein components of phage Mu.

| Gene product | Protein | Amino acid length | Number of copies | Residues | |
|---|---|---|---|---|---|
| | | | | Extended | Contracted |
| **gp29** | Portal | 512 | 12 | 1-304, 322-388 | 1-304, 322-400 |
| **gp34** | Major capsid | 305 | 415 | 1-305 | / |
| **gp36** | Adaptor | 141 | 12 | 1-141 | 1-141 |
| **gp37** | Terminator | 182 | 6 | 1-174 | 1-176 |
| **gp39** | Sheath | 495 | 180 | 2-495 | 2-495 |
| **gp40** | Tube | 118 | 180 | 1-118 | 1-118 |
| **gp42** | Tape measure | 690 | 3 | 674-690 | / |
| **gp43** | Tube initiator | 495 | 6 | 23-278, 282-363<br>383-494 | / |
| **gp44** | Hub | 379 | 3 | 3-347, 357-377 | / |
| **gp45** | Spike | 197 | 3 | 3-197 | / |
| **gp46** | BW1 | 145 | 6 | 2-144 | / |
| **gp47** | BW2 | 360 | 12 | 2-360 | / |
| **gp48** | BW3 | 180 | 6 | 4-177 | / |
| **gp49** | Fiber | 504 | 18 | 1-30, 34-118 | / |
